# Sustainable Land Use Enhances Soil Microbial Respiration Responses to Experimental Heat Stress

**DOI:** 10.1111/gcb.70214

**Published:** 2025-04-24

**Authors:** Rémy Beugnon, Nico Eisenhauer, Alfred Lochner, Margarete J. Blechinger, Paula E. Buhr, Simone Cesarz, Monica A. Farfan, Olga Ferlian, Amanda J. Rompeltien Howard, Yuanyuan Huang, Blanca S. Kuhlmann, Nora Lienicke, Selma Mählmann, Anneke Nowka, Emanuel Petereit, Christian Ristok, Martin Schädler, Jonas T. M. Schmid, Lara J. Schulte, Kora‐Lene Seim, Lise Thouvenot, Raphael Tremmel, Lara Weber, Jule Weitowitz, Huimin Yi, Marie Sünnemann

**Affiliations:** ^1^ German Centre for Integrative Biodiversity Research (iDiv) Halle‐Jena‐Leipzig Leipzig Germany; ^2^ Leipzig Institute of Biology Universität Leipzig Leipzig Germany; ^3^ CEFE, University Montpellier, CNRS, EPHE, IRD Montpellier France; ^4^ Department of Community Ecology Helmholtz‐Centre for Environmental Research – UFZ Halle Germany; ^5^ Institute of Biology/Geobotany and Botanical Garden Martin Luther University Halle‐Wittenberg Halle Germany

**Keywords:** climate change adaptation, global change, land‐use intensity, microbial stress response, soil ecosystem functioning, soil microbial activity

## Abstract

Soil microbial communities provide numerous ecosystem functions, such as nutrient cycling, decomposition, and carbon storage. However, global change, including land‐use and climate changes, affects soil microbial communities and activity. As extreme weather events (e.g., heatwaves) tend to increase in magnitude and frequency, we investigated the effects of heat stress on the activity (e.g., respiration) of soil microbial communities that had experienced four different long‐term land‐use intensity treatments (ranging from extensive grassland and intensive grassland to organic and conventional croplands) and two climate conditions (ambient vs. predicted future climate). We hypothesized that both intensive land use and future climate conditions would reduce soil microbial respiration (H1) and that experimental heat stress would increase microbial respiration (H2). However, this increase would be less pronounced in soils with a long‐term history of high‐intensity land use and future climate conditions (H3), and soils with a higher fungal‐to‐bacterial ratio would show a more moderate response to warming (H4). Our study showed that soil microbial respiration was reduced under high land‐use intensity (i.e., −43% between extensive grassland and conventional cropland) and future climate conditions (−12% in comparison to the ambient climate). Moreover, heat stress increased overall microbial respiration (+17% per 1°C increase), while increasing land‐use intensity reduced the strength of this response (−25% slope reduction). In addition, increasing soil microbial biomass and fungal‐to‐bacterial ratio under low‐intensity land use (i.e., extensive grassland) enhanced the microbial respiration response to heat stress. These findings show that intensive land use and climate change may compromise the activity of soil microbial communities as well as their respiration under heatwaves. In particular, soil microbial communities under high‐intensity land use and future climate are less able to respond to additional stress, such as heatwaves, potentially threatening the critical ecosystem functions driven by soil microbes and highlighting the benefits of more sustainable agricultural practices.

## Graphical abstract

Soil microbes are crucial for healthy ecosystems, but climate change and intensive farming may reduce their functioning. We tested how heat stress affects microbial respiration in soils under different farming practices and climate conditions. Results showed that intensive land use and future climate both reduced microbial respiration. Heatwaves generally increased respiration, but soils from intensive systems responded less. In addition, soils with more fungi and under less intensive use had a stronger response to the stress. These findings suggest that sustainable land use can help soil microbes stay active under climate stress, protecting essential ecosystem functions like nutrient cycling and carbon storage.

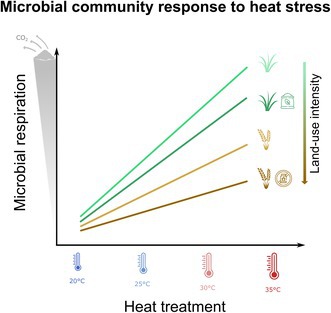

## 1 Introduction

Soil microbial communities play key roles in ecosystems, which is why they have been referred to as the “functional backbones” of terrestrial ecosystems (Van Der Heijden et al. 2008). Composed of predominantly fungi and bacteria, soil microbial communities contribute to critical ecosystem functions and services, such as nutrient cycling, organic matter decomposition, and carbon storage (Coban et al. 2022; Van Der Heijden et al. 2008). For example, a high diversity of microbes has been shown to enhance belowground functioning, improving the decomposition of both labile and recalcitrant carbon sources (Beugnon et al. 2021; Liu et al. 2018; Karhu et al. 2014). Both bacteria and fungi contribute significantly—but differently—to these biogeochemical processes (Wagg et al. 2021).

Soil microbial communities and their functions are increasingly threatened by anthropogenic changes, including increasing land‐use intensity and climate change (Rillig et al. 2019; Sünnemann et al. 2021; Zhou et al. 2020). Intensification of agriculture, driven by economic and population growth (Marques et al. 2019), leads to increasing use of fertilizers, pesticides, and tillage, thus soil compaction, putting significant pressure on soil microorganisms (Beylich et al. 2010; Emmerson et al. 2016; Kopittke et al. 2019; Smith et al. 2016). In particular, higher fertilization rates decrease soil microbial activity in croplands due to nitrogen accumulation over time, leading to a reduction in microbial biomass and respiration, particularly for the fungi, thus causing slower CO_2_ fluxes (Treseder 2008). Additionally, large doses of fertilizers affect both the diversity of bacterial communities as well as the community's ability to efficiently metabolize components of soil organic carbon (SOC, Hu et al. 2025; Kong et al. 2024). As a result, intensive agricultural land use leads to a decrease in SOC and soil nutrient storage, changes in soil properties, and biodiversity (Smith et al. 2016). However, microbial activity in these systems may be modulated by changes in soil temperature and water availability caused by climate change (Domeignoz‐Horta et al. 2021). Understanding how soil microbial communities will respond to future climate scenarios and maintain their activity remains a critical knowledge gap (Knight et al. 2024).

Climate change is altering precipitation patterns, increasing mean temperatures, and the frequency of extreme weather events (Mahecha et al. 2022; Pörtner et al. 2021). These changes have significant impacts on soil microbial communities and functions, including microbial respiration—a key indicator of microbial activity and carbon cycling (Lange et al. 2015; Liu 2013). With increasing seasonal extremes, soils face more frequent extreme drought events during summer, possibly leading to an altered soil microbial diversity, community composition, and gene expression related to nutrient cycling and stress resistance (Bei et al. 2023; de Vries et al. 2012; Nguyen et al. 2018). Short‐term heat stress leads to a rapid microbial response (higher respiration) because microbes experience an immediate metabolic boost due to higher enzyme activity (García et al. 2023; Jones et al. 2006; Yang et al. 2023). In contrast, long‐term warming can lead to physiological adaptations, biomass decline, and shifts in microbial community composition that result in lower microbial respiration and activity over time (Allison et al. 2010; Walker et al. 2018). Specifically, long‐term warming can deplete soil organic matter and reduce carbon availability, limiting microbial growth. Further, microbes may shift toward more heat‐tolerant, but less active or slower‐growing taxa. These warmer conditions may favor microbial communities with lower growth efficiency, leading to a decrease in biomass accumulation (Frey et al. 2013). This may cause more carbon to be released into the atmosphere but also an increase in microbial biomass and necromass and thus long‐term carbon storage in soils (Buckeridge et al. 2020; Hu et al. 2025; Lange et al. 2015; Tao et al. 2023). However, such warming‐induced increases in microbial respiration may not be as large as previously thought (Bradford et al. 2008) and, typically, increase only in the short term (Dacal et al. 2019; Fanin et al. 2022; Hartley et al. 2007). Moreover, soil microbial communities' ability to buffer climate change impacts by enhancing carbon and nutrient cycling and supporting ecosystem stability (Delgado‐Baquerizo et al. 2017; Jansson et al. 2023) depends on the resilience and composition of the microbial communities in response to extreme climate events (de Vries et al. 2012). To fully understand microbial community dynamics in a changing world, it is essential to disentangle two interrelated processes: on the one hand, the long‐term effects of climate change and land‐use intensity on microbial communities and their functioning (e.g., Sünnemann et al. 2021), and on the other hand, the instant responses of these communities to short‐term weather events like heatwaves (e.g., Jones et al. 2006). To address this research gap, we focus on microbial communities that have already been stressed by long‐term global change drivers—because a key knowledge gap lies in understanding how these pre‐stressed communities respond to additional short‐term events (Martínez‐De León and Thakur 2024).

Differences in the microbial community composition (fungal‐to‐bacterial ratio) caused by land‐use intensity (de Vries et al. 2012; Sünnemann et al. 2021) can affect microbial respiration responses to temperature increase (Sáez‐Sandino et al. 2023). For example, studies found that soil microbial communities are less resistant to climate change in intensively used croplands compared to grasslands (Bei et al. 2023; de Vries et al. 2012; Sünnemann et al. 2023). Similarly, low land‐use intensity was shown to increase the fungal‐to‐bacterial ratio (de Vries et al. 2012; Sünnemann et al. 2021), potentially enhancing microbial resistance to warming (de Vries et al. 2012). In such a scenario, fungi likely outperform bacteria in nutrient and water uptake due to their extensive hyphal network (Guhr et al. 2015). Thus, soil microbial communities from intensive croplands, which are mainly dominated by bacterial‐based food webs, are expected to be less resistant to extreme climate events than microbial communities from extensive grasslands that are mainly dominated by fungi‐based food webs due to fungal associations with grasses, forage legumes, and forbs (Canarini et al. 2024; de Vries et al. 2012). However, the effects of land‐use intensity and future climatic conditions on the microbial responses to extreme temperatures remain poorly understood.

**FIGURE 1:**
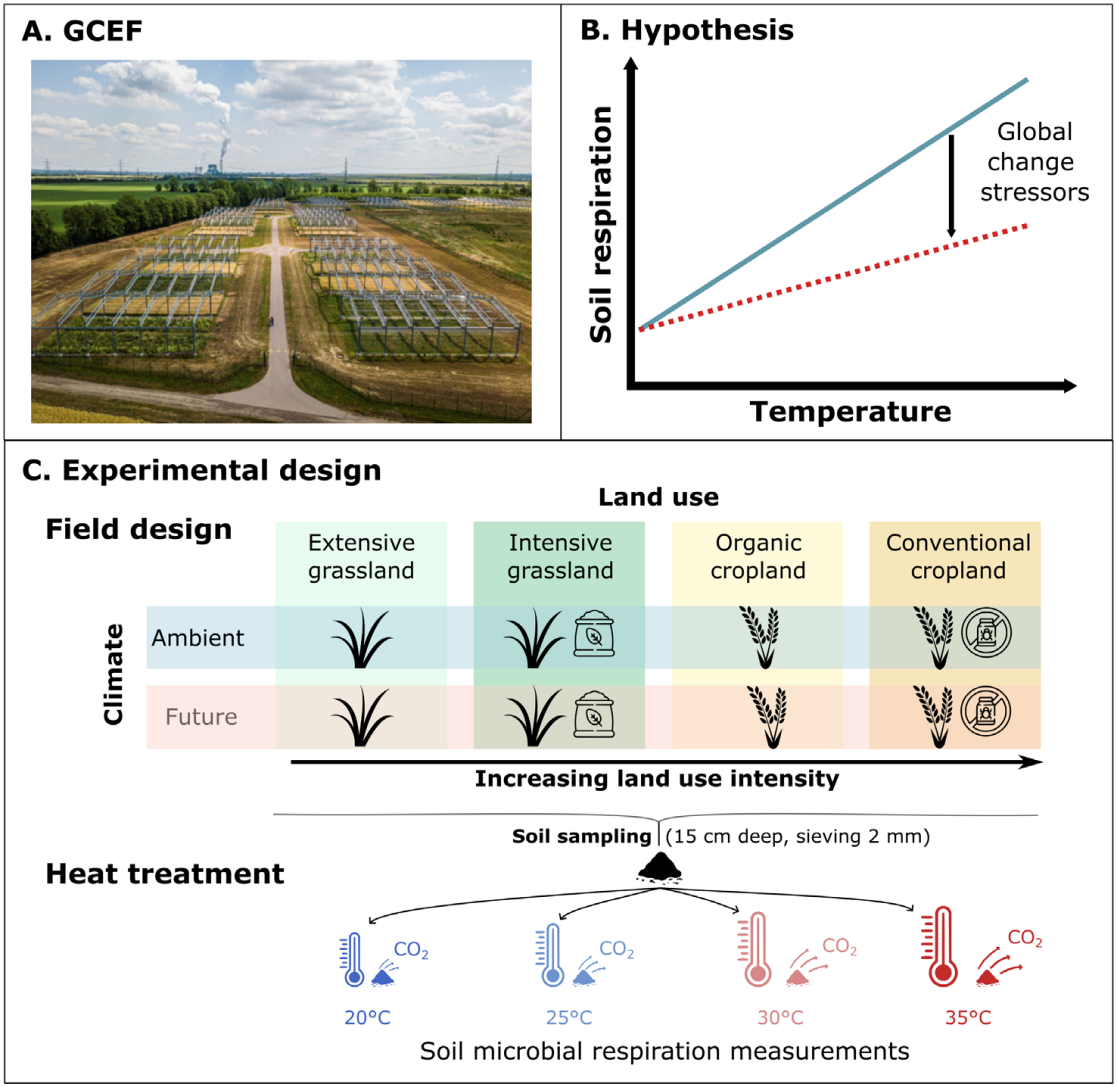
Experimental design and hypothesis. (A) Aerial view of the Global Change Experimental Facility (GCEF, source Künzelmann/UFZ). (B) Expected effect of warming on soil microbial respiration in combination with additional global change drivers. Higher land‐use intensity and climate change are expected to decrease soil microbial activity as indicated by soil microbial respiration response to warming. (C) Experimental design: Full factorial design of the GCEF field experiment and heat treatment established under controlled lab conditions.

Here, using soils from a long‐term field experiment and laboratory heat stress, we investigate the combined history effects of climate change and land‐use intensity on soil microbial respiration and its respiration response to heat stress (Figure 1). Soil samples were collected from the Global Change Experimental Facility (GCEF, Figure 1A), where soils had been subjected to a future climate treatment and varying levels of land‐use intensity for 10 years. To simulate heat stress, soils were incubated at either 20°C, 25°C, 30°C, or 35°C under laboratory conditions, and we assessed the soil microbial respiration response (Figure 1C).

We hypothesized that the long‐term field treatments of future climate and intensive land use would reduce soil microbial respiration due to chronic stress exposure, resulting in lower microbial biomass and potential shifts toward less active microbial communities (H1). Additionally, we expected that, in general, increasing incubation temperature would lead to an increase in microbial respiration, as temperature typically accelerates microbial metabolism under optimal conditions (H2). However, this positive temperature response would be dampened in soils with a long‐term history of future climate and intensive land use (H3) since these microbial communities may be less responsive due to accumulated ecological stress, reduced biomass, or physiological adaptation to recurring extremes (Dacal et al. 2019; Hartley et al. 2007; Martínez‐De León and Thakur 2024). Lastly, we hypothesized that the variation in microbial respiration responses would be mediated by microbial community structure—specifically, a higher fungal‐to‐bacterial ratio would buffer microbial respiration under heat stress due to the capacity of fungi to maintain activity under dry and warm conditions (H4; de Vries et al. 2012; Malik et al. 2016).

## 2 Materials and Methods

To investigate how soil microbial communities respond to short‐term climatic extremes under realistic background conditions, we applied a controlled heat stress treatment on top of an existing long‐term global change experiment. The experiment has been running for over a decade and includes a future climate manipulation (+0.5°C warming, altered precipitation patterns with a summer drought) combined with different land‐use regimes. Importantly, these treatments are superimposed on ambient environmental conditions, meaning that natural climate variability and extreme events such as heatwaves have occurred throughout the duration of the experiment (Bei et al. 2023). As a result, the microbial communities have been shaped by both the experimental treatments and naturally occurring events, creating a realistic baseline of chronic global change exposure. The additional heat stress treatment was applied to simulate an acute short‐term stressor, enabling us to assess microbial responses to extreme temperature events in the context of pre‐existing long‐term stress. This approach reflects future climate scenarios, where ecosystems will be increasingly exposed to compound stressors (IPCC 2021; Pascual et al. 2022).

### 2.1 Study Site

The GCEF is located in Bad Lauchstädt, Central Germany (51°23′30′′ N, 11°52′49′′ E, 116 m a.s.l.) and is part of the Helmholtz Centre for Environmental Research—UFZ (Schädler et al. 2019). The GCEF was established in 2013 on a former arable field to study the influence of climate change on terrestrial ecosystems within different land‐use intensities (Figure 1A). The region is characterized by a sub‐continental climate with an average precipitation of 442 mm and a mean temperature of 10.9°C (2014–2024). The soil type is Haplic Chernozem, which contains 70% silt and 20% clay. Therefore, it holds high levels of organic carbon and has a high water‐holding capacity (Altermann et al. 2005; Korell et al. 2024).

### 2.2 Experimental Setup and Land‐Use Treatments

The GCEF consists of 10 main plots (80 m × 24 m), each divided into 5 plots (16 m × 24 m), resulting in a total of 50 plots. In each main plot, five land‐use treatments were randomly assigned to experimental plots: extensively used grassland, intensively used grassland, organic cropland, conventional cropland, and an extensively used pasture treatment which was excluded from this study to balance the cropland versus grassland treatments. The treatments vary in management intensity, including differences in fertilization, pesticide use, plant species richness, and management practices. Extensive grasslands, representing the lowest management intensity, consist of 56 plant species, receive no fertilization, and are mown twice per year. Intensive grasslands, by contrast, are composed of five grass cultivars, are fertilized with nitrogen (N) and phosphorus (P), and are mown up to four times per year. Organic cropland is managed without any pesticides and includes a 6‐year crop rotation, with legumes sown every 3 years, along with potassium–magnesium–sulfur (K–Mg–S) fertilization. Conventional cropland, the most intensive management type, follows a 3‐year crop rotation of winter rape, winter wheat, and winter barley and relies on the use of N–P–K fertilizers and pesticides (Schädler et al. 2019).

### 2.3 Future Climate Treatment

Half of the plots are exposed to a future climate scenario, whereas the other half is exposed to ambient climate conditions. The future climate scenario was designed based on regional climate models for 2070–2100: It features a general temperature increase of 0.55°C, a 10% increase in precipitation during spring and fall, and a 20% decrease in precipitation during summer (Schädler et al. 2019). The climate manipulation was achieved by steel roof structures above each main plot, entailing tarpaulins that are closed from sunset to sunrise to achieve passive nighttime warming (Figure S1). Additionally, the tarpaulins also reduce rainfall in summer, while an irrigation system is used to increase precipitation. Control plots had similar roof constructions to account for potential side effects (Kreyling et al. 2017).

### 2.4 Soil Sampling

Soil samples were collected on October 10, 2024, using a steel core sampler with a diameter of 1.5 cm and a depth of 15 cm. To account for potential heterogeneity, five subsamples were taken per plot, pooled, and sieved through a 2 mm mesh. The resulting soil samples were then used to measure soil microbial respiration at four different soil temperatures, with two separate measurements conducted (i.e., technical replicates) to ensure reliable results. For the first measurement, the samples were stored at 4°C. For the second measurement, the samples were frozen at −20°C to preserve them for later analysis. Prior to soil microbial respiration and community analysis, unfrozen samples were acclimated at 20°C for 3 days, while frozen samples were acclimated for 7 days at 20°C to ensure complete defrosting.

### 2.5 Soil Microbial Analyses and Heat Treatment

Soil microbial respiration was measured using an O_2_‐micro‐compensation system (Scheu 1992). For each soil sample, four subsamples (approximately 7 g) were collected. Each subsample was subjected to one out of the four temperature conditions—20°C, 25°C, 30°C, or 35°C—for a duration of 20 h while measuring soil microbial respiration as the oxygen consumption per hour per dry weight of soil in microliter O_2_ per gram dry weight per hour for a 24‐h interval (Table S1). To assess total soil microbial biomass (μg C_mic_ g^−1^ soil dry weight), we measured the maximum respiratory response by adding glucose (4 mg g^−1^ dry weight soil, dissolved in 1.25 mL distilled water) to soil samples measured at 20°C (following Scheu 1992). This approach provides a robust proxy for the size of the active microbial biomass. We did not correct samples for soil moisture content across treatments as soils were supposed to reflect natural field conditions under different long‐term treatments. We acknowledge that differences in initial moisture content may influence microbial respiration, but these were considered an integral part of the treatment effect and not experimentally standardized.

We used phospholipid fatty acid (PLFA) analysis to assess microbial community structure, as it provides quantitative measurements of living microbial biomass and major functional groups (e.g., Gram‐positive/Gram‐negative bacteria, fungi). This method captures functionally relevant shifts that are closely linked to microbial respiration, offering a more direct connection to ecosystem processes such as respiration than DNA‐based approaches, which may include relic DNA and inactive community members (Carini et al. 2016). We followed the methodology described by Frostegård et al. (1991), using 5 g of fresh soil per sample. Fatty acid methyl esters were analysed on a gas chromatograph (as described in Cesarz et al. 2023). We used FA 19:0 as an internal standard to quantify bacterial and fungal PLFAs and neutral lipid fatty acids (NLFAs) in ng fatty acid methyl ester (FAME) g^−1^ soil and to assign them to microbial groups. Bacteria were grouped in Gram‐positive bacteria (PLFAs a15:0, i15:0, i16:0, and i17:0), Gram‐negative (cy17:0, cy19:0), and widespread bacteria (16:1ω7), while fungi were grouped in arbuscular mycorrhizal fungi (NLFA 16:1ω5) and saprotrophic and ectomycorrhizal fungi (PLFA 18:2ω6,9, Ruess and Chamberlain 2010). Finally, the fungal‐to‐bacterial ratio was calculated by dividing the sum of all fungi‐specific PLFAs and NLFAs by the sum of all bacteria‐specific PLFAs.

### 2.6 Statistical Analyses

All data handling and statistical analyses were performed using the R software (version 4.4.2.). R scripts used for this project can be found in Supporting Information S1–S4. All of the following linear mixed‐effect models were tested using the lmer function from the ‘lme4’ package (Bates et al. 2015), and statistical hypotheses (i.e., residuals normality, homoscedasticity) of the following linear models were tested using the model_check function from the ‘performance’ package (Lüdecke et al. 2020).

#### 2.6.1 Land Use and Climate Effects on Soil Respiration

We used linear mixed models and normal distribution assumptions to test the effects of land use (four levels) and climate (two levels) treatments on soil microbial respiration at 20°C. In addition, the experimental sampling plot nested within the main plot (Schädler et al. 2019) and the respiration measurement time (unfrozen vs. frozen samples) were set as random factors. Soil microbial respiration was log‐transformed to fulfill statistical assumptions. Our experimental data were completely orthogonal and free of missing values. The significance of the explanatory variables was tested using an ANOVA type I. In addition, a post hoc test was performed to test the differences between land‐use levels using the glht function from the ‘multcomp’ package (Hothorn et al. 2002).

#### 2.6.2 Land Use and Climate Effects on Soil Respiration Response to Heat Treatment

Similarly, we used linear mixed models and normal distribution assumptions to test the effects of heat treatment (as a linear temperature variable), land use, climate, and their interactions on soil microbial respiration. The same random terms (sampling plot nested within the main plot and the respiration measurement time) were applied, and the soil microbial respiration was log‐transformed to fulfill statistical assumptions. The significance of the explanatory variables was tested using an ANOVA type I.

#### 2.6.3 Land Use and Climate Effects on Soil Microbial Biomass and Community Composition

We used linear mixed models and normal distribution assumptions to test the effects of land use, climate, and their interaction on soil microbial community composition, namely microbial biomass (based on substrate‐induced respiration), fungal biomass, bacterial biomass, and fungal‐to‐bacterial ratio. Fungal biomass, bacterial biomass, and fungal‐to‐bacterial ratio were log‐transformed to fulfill statistical assumptions. The sampling main plot was set as a random factor. The significance of the explanatory variables was tested using an ANOVA type I. In addition, a post hoc test was performed to test the differences between land‐use levels using the glht function from the ‘multcomp’ package.

## 3 Results

### 3.1 Land Use and Climate Effects on Soil Microbial Respiration

**FIGURE 2:**
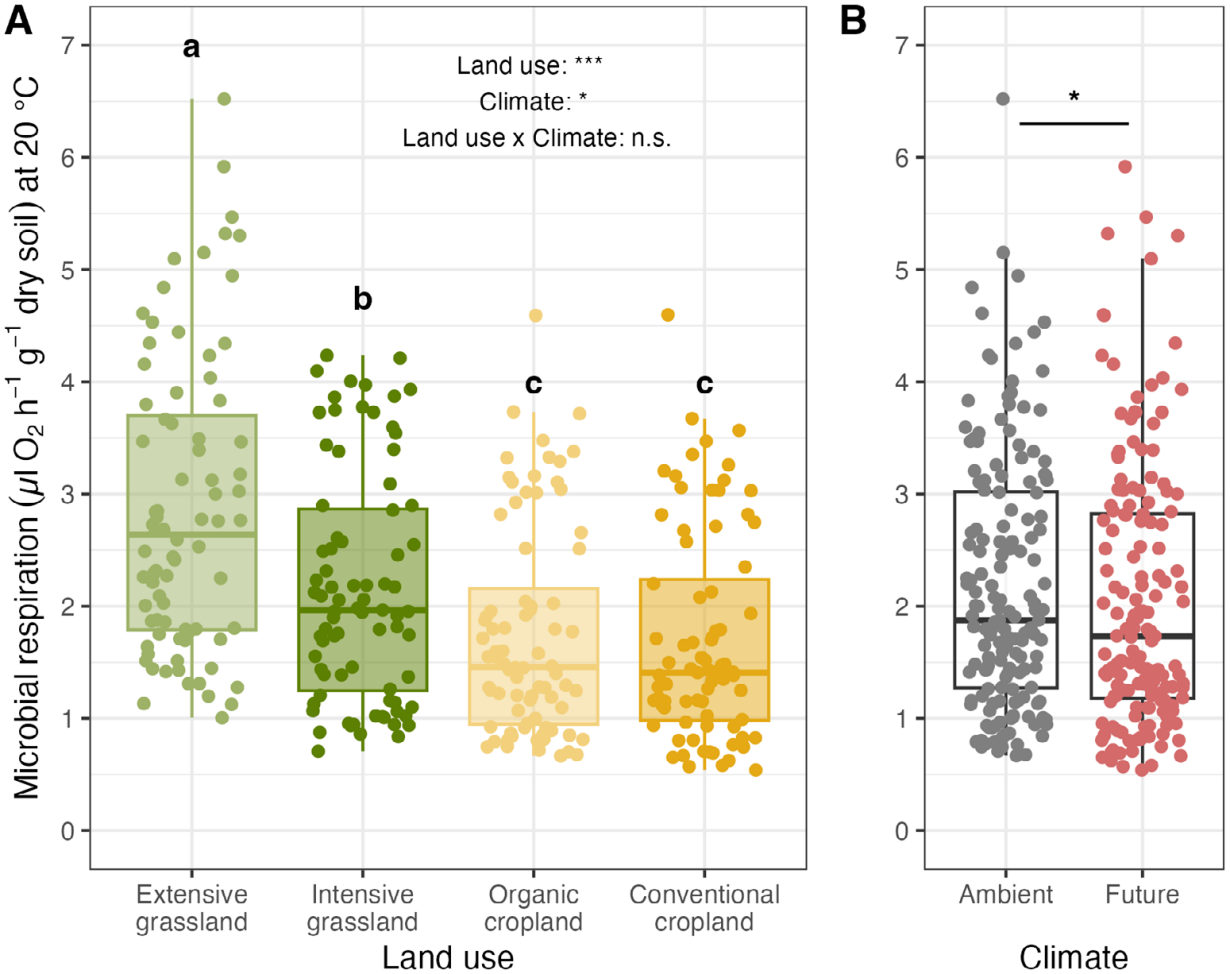
Land use (A) and future climate (B) effects on soil microbial respiration (H1). Colors depict the different land‐use (A) and climate (B) treatments; in addition, points were jittered for better visual assessment. Different letters indicate statistically different means based on a Holm‐adjusted post hoc test (*p* < 0.05). Significance levels were standardized: *p* > 0.1: n.s., *p* < 0.1: (*), *p < 0.05, **p < 0.01, ***p < 0.001. Note that respiration values were log‐transformed in the statistical model to fulfill statistical assumptions; see Supplementary Material S1 and Figure S2.

Soil microbial respiration decreased significantly with increasing land‐use intensity (*F*_3,24_ = 100.3, *p* < 0.001) and under future climate conditions (−6%; *F*_1,8_: 8.07, *p* = 0.02; Figure 2; Supporting Information S1). More specifically, grasslands exhibited higher soil microbial respiration than croplands (+40%, Figure 2A); within grasslands, extensive grasslands had significantly higher microbial respiration than intensive grasslands (+32%, *p* < 0.001, Figure 2A). In contrast, the two cropland treatments did not differ significantly from each other (*p* > 0.05). Notably, land‐use intensity and future climate treatment did not show any significant interactive effects on soil microbial respiration (*F*_3,24_ = 2.18; *p* = 0.101).

### 3.2 Soil Microbial Respiration Response to Heat Treatment Under Contrasting Land‐Use Conditions

**FIGURE 3:**
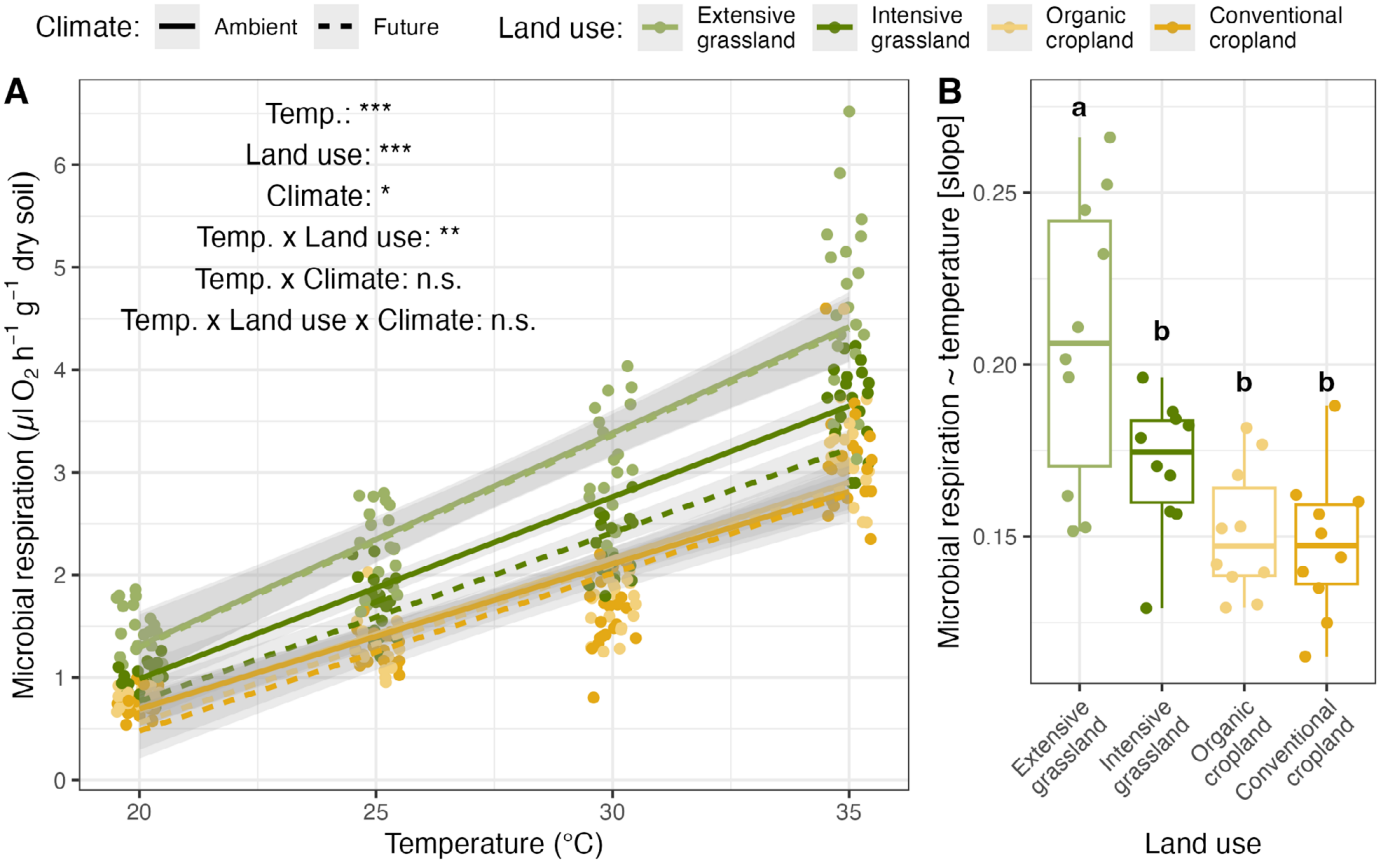
Soil microbial respiration responses (H2) to simulated heat stress (A) across climate and land‐use intensity treatments (B). (A) Microbial respiration–temperature relationship under contrasting land‐use intensity and climate treatments. Line colors represent the different land‐use intensities, while the line types depict the climate treatments (full: “Ambient” vs. dashed: “Future”). Significance levels were standardized: *p* > 0.1: n.s., *p* < 0.1: (*), *p < 0.05, **p < 0.01, ***p < 0.001. (B) Land‐use intensity effect of the relationship between soil microbial respiration and simulated heat, i.e., the slope of the linear relationship. Different letters indicate statistically different means based on a Holm‐adjusted post hoc test (*p* < 0.05). Points were jittered for visual assessment. Note that respiration values were log‐transformed in the statistical model to fulfill statistical assumptions; see Supporting Information S2 and Figure S3.

Soil microbial respiration increased significantly with higher temperatures across all land‐use and climate treatments (*F*_1,271_ = 2696.04; *p* < 0.001; Figure 3A; Supporting Information S2). Increasing land‐use intensity (*F*_3,24_ = 22.43; *p* < 0.001) and future climate (*F*_1,8_ = 4.72; *p* = 0.031) decreased microbial respiration across the four heat treatments though (Figure 3A; Supporting Information S2). Furthermore, heat stress and land use showed a significant interaction effect on microbial respiration (*F*_3,271_ = 3.77; *p* = 0.011; Figure 3B), with microbial respiration increasing more under extensive grassland than under intensive land use (i.e., intensive grassland and organic and conventional croplands; Figure 3B). Markedly, when we looked at the proportional scale, looking at the log‐transformed microbial respiration, the slope was shallowest under extensive grassland (Figure S3).

### 3.3 Soil Microbial Community Composition Responses to Land Use and Consequences for Microbial Respiration Response to a Heatwave

**FIGURE 4:**
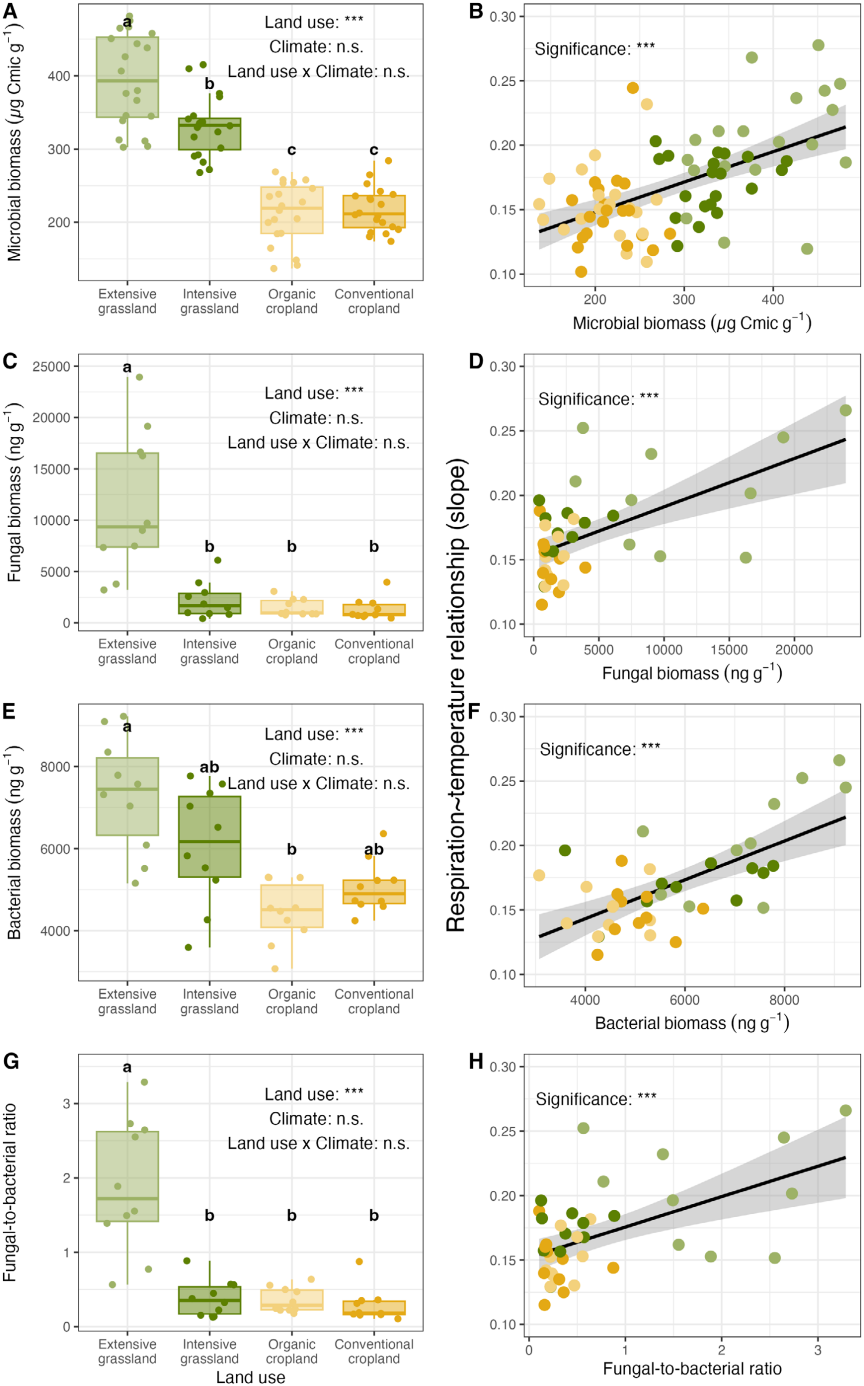
Effects of land use on soil microbial biomass and community properties (A, C, E, G) and relationships between soil microbial community properties and the microbial respiration response to heat stress (B, D, F, H) (H4). Colors represent the different land‐use treatments. In addition, points were jittered for better visual assessment. Different letters indicate statistically different means based on a Holm‐adjusted posthoc test (*p* < 0.05), and significance levels were standardized: *p* > 0.1: n.s., *p* < 0.1: (*), *p < 0.05, **p < 0.01, ***p < 0.001.

We observed higher soil microbial biomass with decreasing land‐use intensity (*F*_3.24_ = 137, *p* < 0.001; Figure 4A; Supporting Information S3). The total soil fungal biomass was significantly higher in extensive grasslands compared to the other land‐use intensity treatments (*p* < 0.001, Figure 4C), while the total soil bacterial biomass in extensive grasslands was only significantly higher than in the organic cropland (*p* < 0.001, Figure 4E). The soil fungal‐to‐bacterial ratio was the highest in extensive grasslands and significantly different from the other land‐use intensity treatments (Figure 4G). However, there were no significant effects of climate as well as of the interaction between land use and climate on microbial biomass, fungal biomass, bacterial biomass, and fungal‐to‐bacterial ratio (both *p* > 0.05, Supporting Information S3).

Temperature sensitivity of soil microbial respiration increased with increasing total soil microbial biomass ($R_{\text{marginal}}^{2}=34\%$, *p* < 0.001; Figure 4B), fungal biomass ($R_{\text{marginal}}^{2}=36\%$, *p* < 0.001; Figure 4D), bacterial biomass ($R_{\text{marginal}}^{2}=42\%$, *p* < 0.001; Figure 4F), and increasing fungal‐to‐bacterial ratio ($R_{\text{marginal}}^{2}=31\%$, *p* < 0.001; Figure 4H; Supporting Information S4).

## 4 Discussion

We examined the interactive effects of long‐term land‐use intensity and climate change, as well as heat stress under laboratory conditions, on soil microbial respiration. Our findings show that both land‐use intensity and future climate conditions reduce soil microbial respiration. However, while experimental heat stress increased microbial respiration, this positive effect was weakened under intensive land‐use practices, such as croplands and intensive grasslands. Overall, soil microbial biomass and the fungal‐to‐bacterial ratio were higher in extensive land‐use systems, such as extensive grasslands, which also amplified the heat stress sensitivity of soil microbial respiration. These findings indicate that intensive land use and climate change compromise soil microbial respiration and biomass during extreme climate events. Moreover, microbial functions are better equipped to cope with heat stress when soils are managed under sustainable practices.

We found an overall negative effect of future climate conditions on soil microbial respiration. However, soils exposed to future climate treatments in the field for the past 10 years exhibited a diminished microbial respiration response to experimental heat stress. This suggests that chronic stress, such as prolonged exposure to elevated temperatures and altered precipitation patterns, may outweigh the adaptive processes of soil microbes (Bérard et al. 2015). In particular, the future climate treatment, including enhanced past extreme events (e.g., summer 2018–2019, Figure S3), might have increased the ecological debt (Martínez‐De León and Thakur 2024), limiting the ability of the communities to face future extreme events such as heatwaves. Future climate conditions may affect soil microbes through two contrasting mechanisms: increased temperatures which typically enhance microbial respiration and reduced summer precipitation which lowers soil water content and suppresses microbial respiration (Curiel Yuste et al. 2007). The interplay of these factors resulted in a net negative impact on soil microbial respiration (Treseder 2008). This aligns with previous results highlighting the non‐additive detrimental effects of increased temperature and reduced summer precipitation on soil biological activity (Thakur et al. 2018). However, we did not observe any significant effect of future climate conditions on soil microbial community composition, as previously shown in a GCEF study (Sünnemann et al. 2021). This indicates that the physiological responses to the future climate are not driven by differences in broad microbial taxonomic groups or their physiology (Manzoni et al. 2012). However, PLFA analyses quantify only major microbial taxa and do not capture species‐level fluctuations or the expression of functional genes, which also influence soil microbial community functioning (Bei et al. 2023; Trivedi et al. 2016) and carbon‐use efficiency (Hu et al. 2025). To better understand specific physiological responses, additional analyses such as metabolic profiling are needed.

Our results confirm that intensive land use practices diminish microbial responsiveness to heat stress, consistent with earlier findings at this site (Siebert et al. 2019; Sünnemann et al. 2021, 2023). These findings suggest that soil microbial communities under high‐intensity land use and future climate conditions are less able to respond to additional stressors such as heatwaves. Several potential mechanisms may explain this pattern. Intensive practices, such as frequent mowing and tillage, degrade soil structure by causing compaction and disrupting soil aggregates. This reduces oxygen availability, carbon concentrations, and water retention (Bei et al. 2023; Greenland 1977). These unfavorable conditions limit microbial habitats, restrict access to substrates, and reduce organic material in soil, thereby diminishing energy sources for microbes (He et al. 2024; Liang et al. 2021). Furthermore, intensive land use tends to promote functionally less diverse microbial communities and shifts toward stress‐tolerant taxa, such as Actinobacteria, which are typically less metabolically active and less responsive to environmental fluctuations (Bei et al. 2023). These structural changes in the microbial community likely underlie the diminished microbial respiration response to the experimental heat stress, as microbial community composition strongly influences ecosystem functioning (Bei et al. 2023; Tardy et al. 2015).

In contrast, extensive grasslands supported higher microbial biomass and fungal‐to‐bacterial ratios, which increased the sensitivity of soil microbial respiration to heat stress. This suggests that fungal‐dominated communities can maintain physiological activity during heat stress. Minimal soil disturbance (Strickland and Rousk 2010), lack of fertilization (Ramirez et al. 2012), and high plant diversity likely enhanced microbial activity and fungal biomass (Bais et al. 2006; de Vries et al. 2012; Hu et al. 2025; Tsiafouli et al. 2015). Diverse plant communities provide varied and continuous organic matter inputs, such as root exudates and plant residues, which fuel microbial activity (Eisenhauer et al. 2017) and create favorable conditions for microbial decomposition during heatwaves (Angst et al. 2023). These plant communities also support fungi by offering a broader range of carbon sources, including complex and recalcitrant compounds (Steinauer et al. 2016). Additionally, higher plant diversity contributes to greater plant cover, maintaining soil moisture (Vogel et al. 2013), higher microbial carbon‐use efficiency (Eisenhauer et al. 2013), and thereby promoting resilience against environmental stressors. Interestingly, plant biomass or yield was not directly correlated with microbial or fungal biomass (Figure S4), suggesting that plant diversity, rather than productivity, plays a key role in supporting soil microbial functioning. This highlights the importance of biodiversity‐based management over yield‐maximizing approaches for building resilient microbial communities. However, this study focused on microbial respiration during short‐term heat stress, limiting its comparability to long‐term ecosystem processes. While land use and climate change were simulated over an extended period in the field, the heat stress treatment occurred in a closed system where soil moisture was held constant. As natural heat waves often reduce soil moisture, our lab conditions may underestimate the compounded stress of heat and drought (Borowik and Wyszkowska 2016; Perkins et al. 2015).

Our study indicates that intensive land use and climate change compromise soil microbial biomass and respiration during extreme climate events. More specifically, we confirm the beneficial impact of sustainable land‐use practices on microbial functioning (Siebert et al. 2019; Sünnemann et al. 2021, 2023), highlighting that extensive land use enhances microbial respiration under heat stress, whereas future climate conditions tend to reduce it. This suggests that soils under intensive management may become increasingly vulnerable to environmental stressors. However, our results also point toward tangible mitigation strategies. Promoting extensive or diversified land use, increasing plant diversity, and reducing tillage intensity could help sustain microbial biomass, enhance microbial resilience, and support ecosystem functions under climate stress (Domeignoz‐Horta et al. 2024; Domnariu et al. 2025). While our findings are based on soils from a single long‐term global change experiment with a specific soil type, they align with broader evidence suggesting that sustainable land management can improve the resistance and resilience of soil microbial communities (Sünnemann et al. 2024). To generalize these findings, future research should expand across different soil types, regions, and land‐use systems (Griffiths and Philippot 2013; Knight et al. 2024; Sáez‐Sandino et al. 2023). Moreover, exploring the long‐term impacts of repeated heatwaves, seasonal dynamics, and microbial adaptation processes will be essential to better understand soil functioning in a rapidly changing climate (Bastos et al. 2021; Schnecker et al. 2023; Yu et al. 2018). In the context of the current climate crisis and the rising frequency of extreme weather events, adopting sustainable agricultural practices will be critical to preserve soil microbial functioning and associated ecosystem services such as carbon storage and nutrient cycling (Lange et al. 2015). Proactive land‐use strategies may also help reduce the ecological debt that limits microbial responsiveness to future climatic extremes.

## Author Contributions

**Rémy Beugnon:** conceptualization, data curation, formal analysis, supervision, validation, visualization, writing – original draft. **Nico Eisenhauer:** conceptualization, funding acquisition, supervision, writing – original draft. **Alfred Lochner:** formal analysis, supervision. **Margarete J. Blechinger:** formal analysis, visualization, writing – original draft. **Paula E. Buhr:** formal analysis, investigation, visualization, writing – original draft. **Simone Cesarz:** validation, writing – review and editing. **Monica A. Farfan:** supervision, writing – review and editing. **Olga Ferlian:** supervision, writing – review and editing. **Amanda J. Rompeltien Howard:** investigation, visualization, writing – original draft. **Yuanyuan Huang:** data curation, supervision, writing – review and editing. **Blanca S. Kuhlmann:** investigation, visualization, writing – original draft. **Nora Lienicke:** investigation, visualization, writing – original draft. **Selma Mählmann:** investigation, visualization, writing – original draft. **Anneke Nowka:** investigation, visualization, writing – original draft. **Emanuel Petereit:** investigation, visualization, writing – original draft. **Christian Ristok:** formal analysis, supervision, writing – review and editing. **Martin Schädler:** conceptualization, supervision, writing – review and editing. **Jonas T. M. Schmid:** investigation, visualization, writing – original draft. **Lara J. Schulte:** investigation, visualization, writing – original draft. **Kora‐Lene Seim:** investigation, visualization, writing – original draft. **Lise Thouvenot:** supervision, writing – review and editing. **Raphael Tremmel:** investigation, writing – review and editing. **Lara Weber:** investigation, visualization, writing – original draft. **Jule Weitowitz:** investigation, visualization, writing – original draft. **Huimin Yi:** supervision, writing – review and editing. **Marie Sünnemann:** conceptualization, formal analysis, investigation, supervision, validation, writing – original draft.

## Conflicts of Interest

The authors declare no conflicts of interest.

## Supporting information


Data S1.

## Data Availability

All datasets and codes that support the findings of this study are openly available in our Dryad repository at 10.5061/dryad.f4qrfj76n.
