# Supplementary material for: Sustainable Land Use Enhances Soil Microbial Respiration Responses to Experimental Heat Stress
Source: Glob Chang Biol. 2025 Apr 24;31(4):e70214. doi: 10.1111/gcb.70214 (PMC12020990; doi:10.1111/gcb.70214)

Sustainable land use enhances soil microbial activity responses to experimental heat waves

Rémy Beugnon, Alfred Lochner, Nico Eisenhauer, Margarete J. Blechinger, Paula E. Buhr, Simone Cesa

2025-04-11

Contents

Samples distribution **2**

Supplementary table S1: sample distribution across treatments. Land use treatments: EM (extensive grassland), IG (intensive grassland), OF (organic cropland), and CF (conventional cropland). Climate treatments: A (ambiant climate) and F (future climate). 3

Future climate treatment **5**

Supplementary Figure S1: Climate treatment (Ambient: A vs. Future: F) on soil maximum (A), average (B), and minimum (C) daily temperature at 5 cm depth. Soil temperatures were collected hourly from January 1st to December 31st, 2024. Monthly differences between the treatments were tested using a t-test. p-values were reported using ns. : p > 0.05, * : 0.05 > p > 0.01, ** : 0.01 > p > 0.001, *** : p > 0.001. 6

Figure 2: land use and climate effect on soil microbial respiration **7**

Supplementary material S1: statistical model fit and quality control 7

Supplementary Figure S2: log-transformed respiration 16

Figure 3: temperature treatment interaction with land use and climate **17**

Supplementary material S2: statistical model fit and quality control 17

Supplementary Figure S3: log-transformed respiration 25

Figure 4 **26**

Supplementary material S3: land use and climate effects on soil microbial community 26

Supplementary material S4: soil microbial community effects on response to temperature treatment 37

Supplemantary Figure S4: Relationship between land use, plant biomass production (Yield, T/ha/y), and soil microbial community (total microbial biomass, fungi biomass, bacterial biomass, and fungal-to-bacterial ratio) **45**

Samples distribution

landuse	climate	temperature	replicate	# sample
EM	A	20	1	5
EM	A	20	2	5
EM	A	25	1	5
EM	A	25	2	5
EM	A	30	1	5
EM	A	30	2	5
EM	A	35	1	5
EM	A	35	2	5
EM	F	20	1	5
EM	F	20	2	5
EM	F	25	1	5
EM	F	25	2	5
EM	F	30	1	5
EM	F	30	2	5
EM	F	35	1	5
EM	F	35	2	5
IG	A	20	1	5
IG	A	20	2	5
IG	A	25	1	5
IG	A	25	2	5
IG	A	30	1	5
IG	A	30	2	5
IG	A	35	1	5
IG	A	35	2	5
IG	F	20	1	5
IG	F	20	2	5
IG	F	25	1	5
IG	F	25	2	5
IG	F	30	1	5
IG	F	30	2	5
IG	F	35	1	5
IG	F	35	2	5
OF	A	20	1	5
OF	A	20	2	5
OF	A	25	1	5
OF	A	25	2	5
OF	A	30	1	5
OF	A	30	2	5
OF	A	35	1	5
OF	A	35	2	5
OF	F	20	1	5
OF	F	20	2	5
OF	F	25	1	5
OF	F	25	2	5
OF	F	30	1	5
OF	F	30	2	5
OF	F	35	1	5
OF	F	35	2	5
CF	A	20	1	5
CF	A	20	2	5

landuse	climate	temperature	replicate	# sample
CF	A	25	1	5
CF	A	25	2	5
CF	A	30	1	5
CF	A	30	2	5
CF	A	35	1	5
CF	A	35	2	5
CF	F	20	1	5
CF	F	20	2	5
CF	F	25	1	5
CF	F	25	2	5
CF	F	30	1	5
CF	F	30	2	5
CF	F	35	1	5
CF	F	35	2	5

Supplementary table S1: sample distribution across treatments. Land use treatments: EM (extensive grassland), IG (intensive grassland), OF (organic cropland), and CF (conventional cropland). Climate treatments: A (ambiant climate) and F (future climate).

Future climate treatment

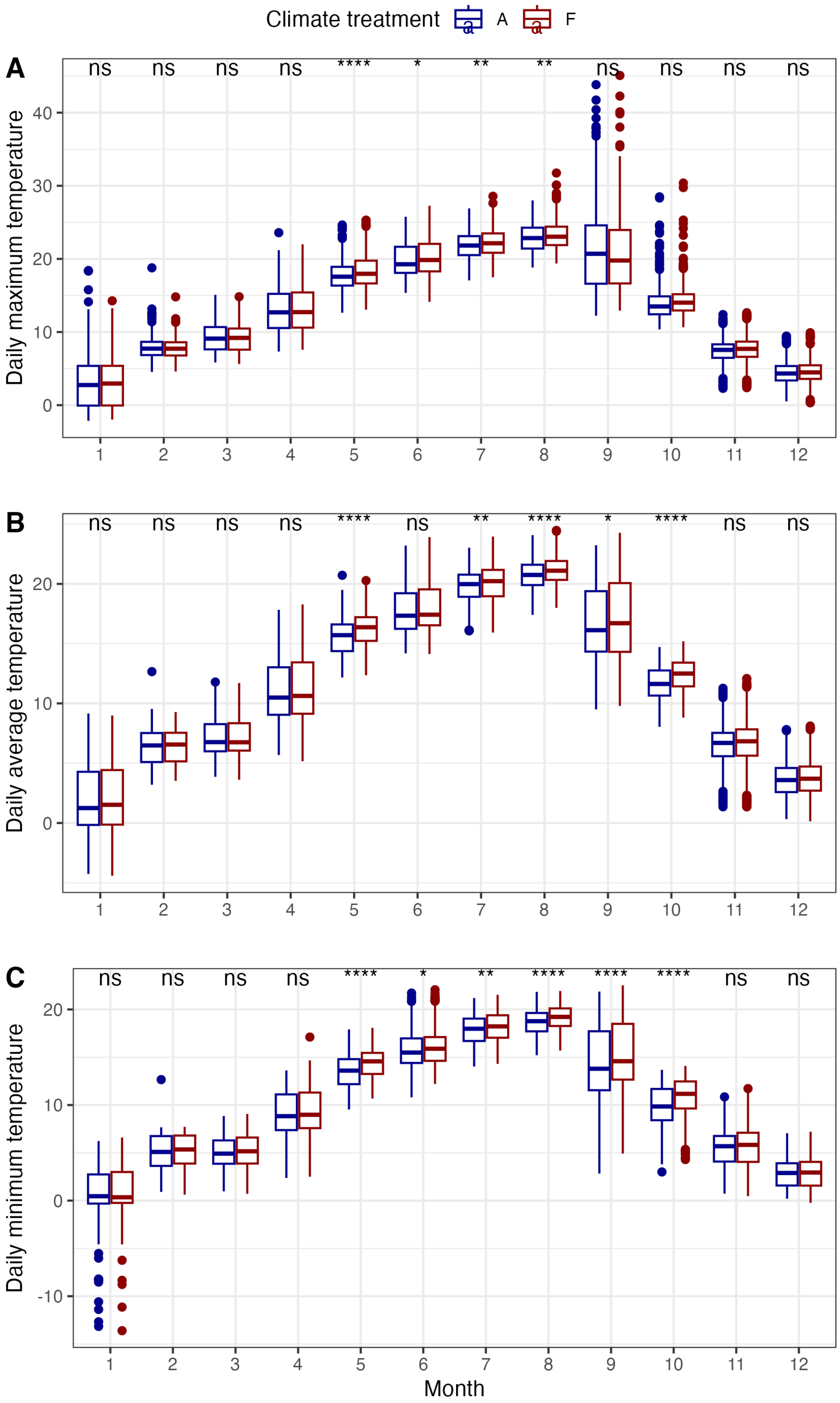

Supplementary Figure S1: Climate treatment (Ambient: A vs. Future: F) on soil maximum (A), average (B), and minimum (C) daily temperature at 5 cm depth. Soil temperatures were collected hourly from January 1st to December 31st, 2024. Monthly differences between the treatments were tested using a t-test. p-values were reported using ns. : $p > 0.05$, * : $0.05 > p > 0.01$, ** : $0.01 > p > 0.001$, *** : $p > 0.001$.

Figure 2: land use and climate effect on soil microbial respiration

Supplementary material S1: statistical model fit and quality control

Statistical model:

```
model = lmer(
  formula = 'log(resp) ~ landuse * climate + (1|mainplot/plot) + (1|rep)',
             data = df |> filter(temperature == 20))
```

Fit quality:

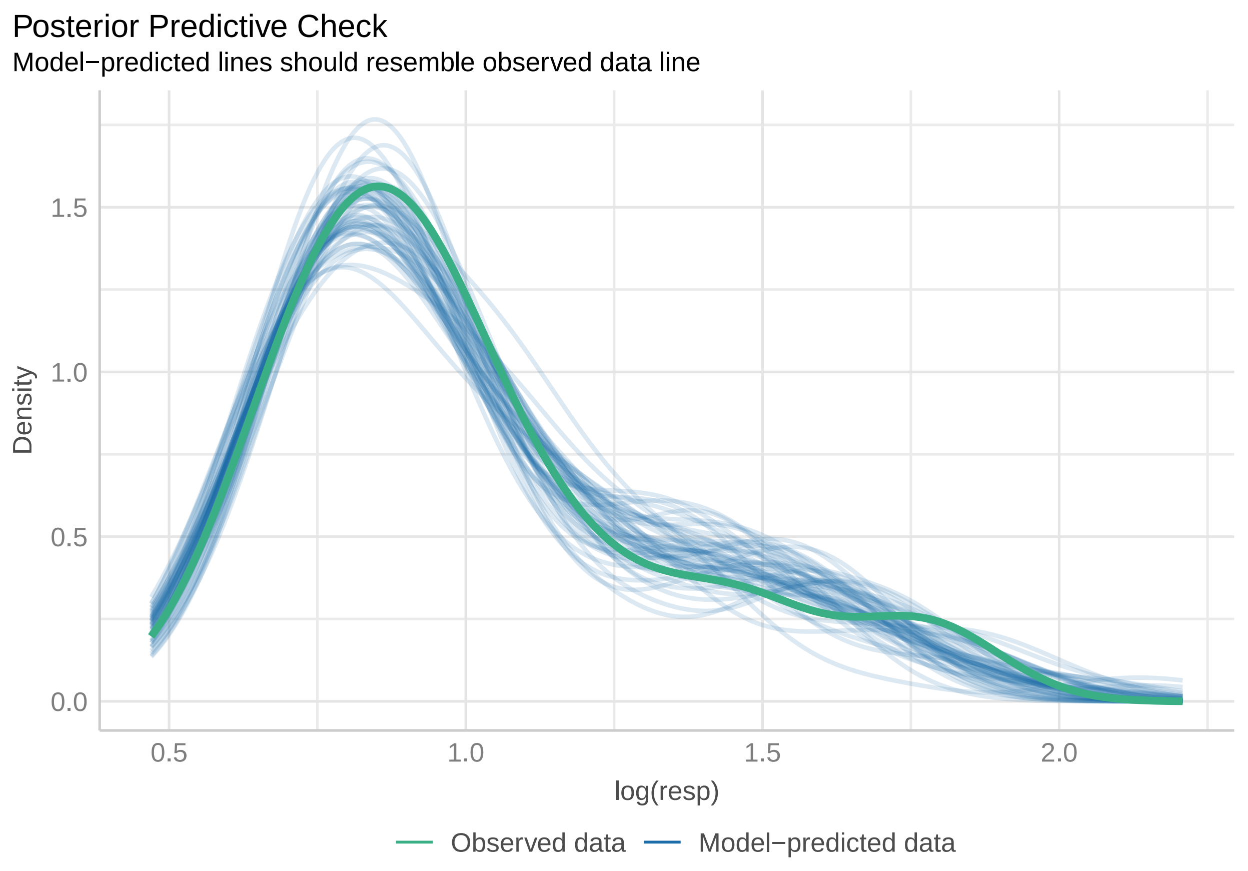

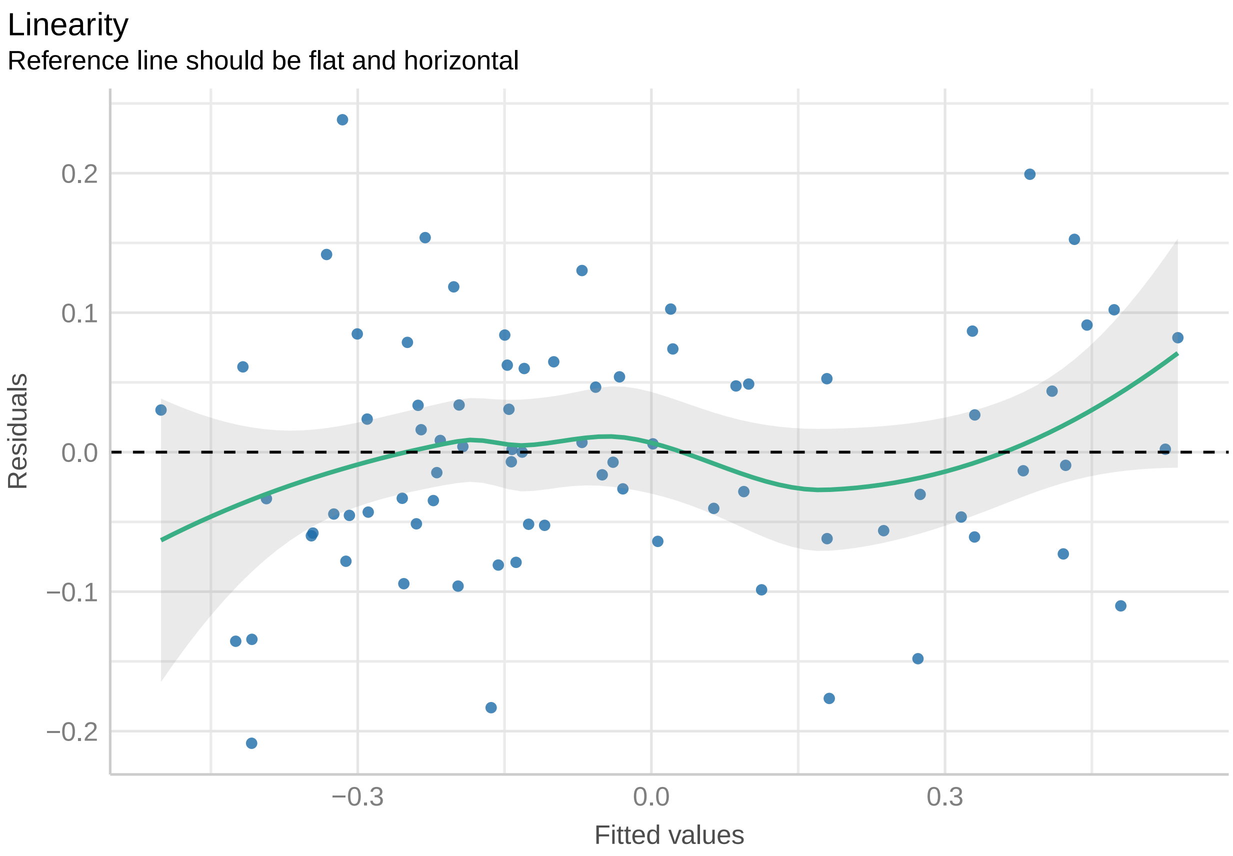
Linearity
Reference line should be flat and horizontal
Residuals
0.2
0.1
0.0
−0.1
−0.2
−0.3
0.0
0.3
Fitted values

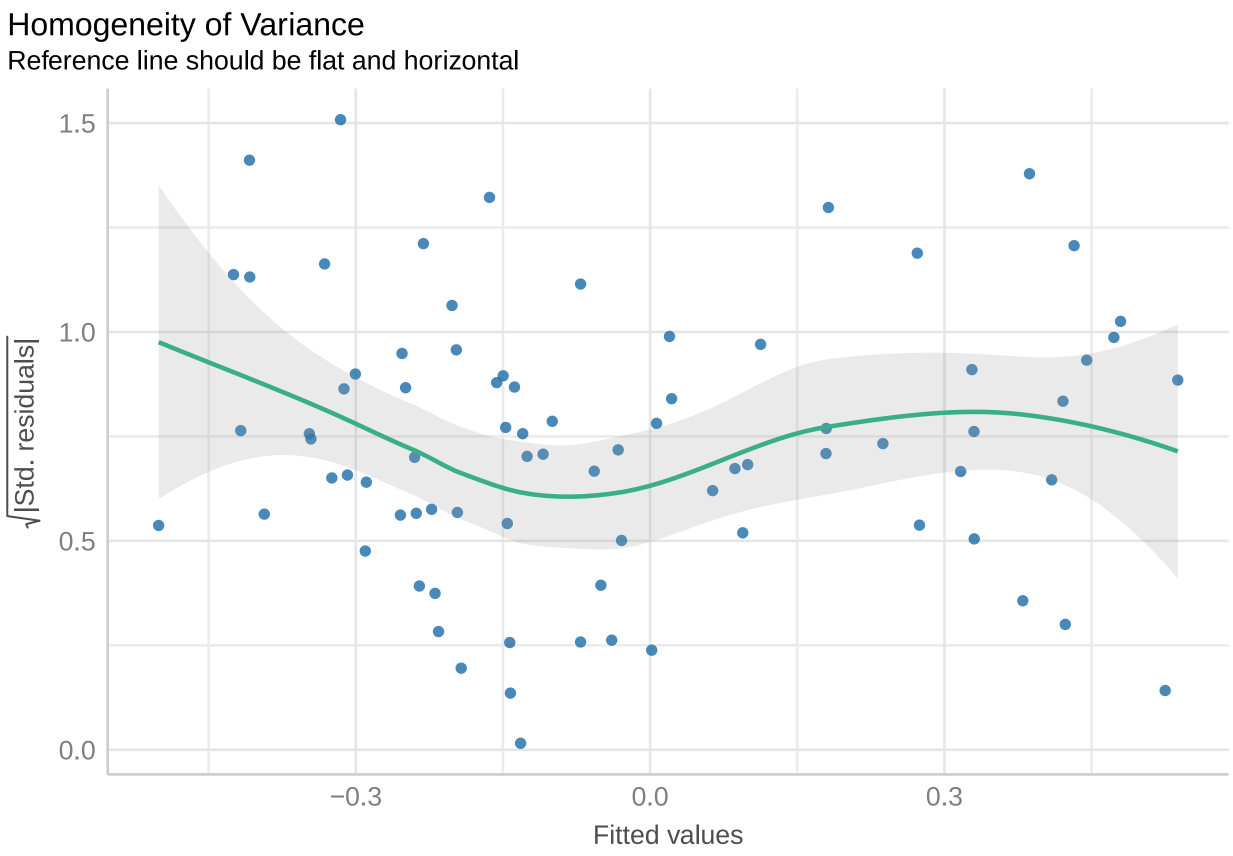
Homogeneity of Variance
Reference line should be flat and horizontal
1.5
1.0
0.5
0.0
√|Std. residuals|
−0.3
0.0
0.3
Fitted values

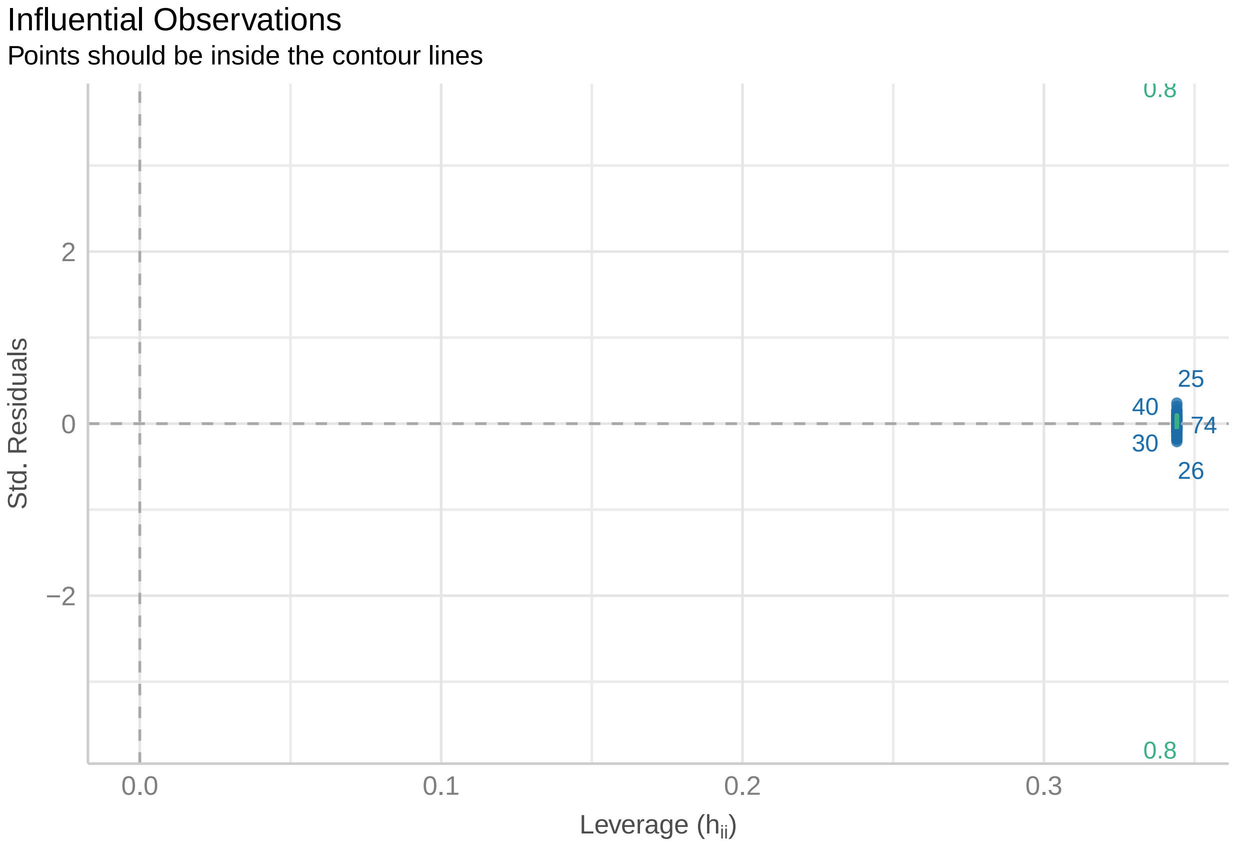
Influential Observations
Points should be inside the contour lines
0.8
2
0
−2
0.8
25
40
74
30
26
Std. Residuals
0.0
0.1
0.2
0.3
Leverage (hii)

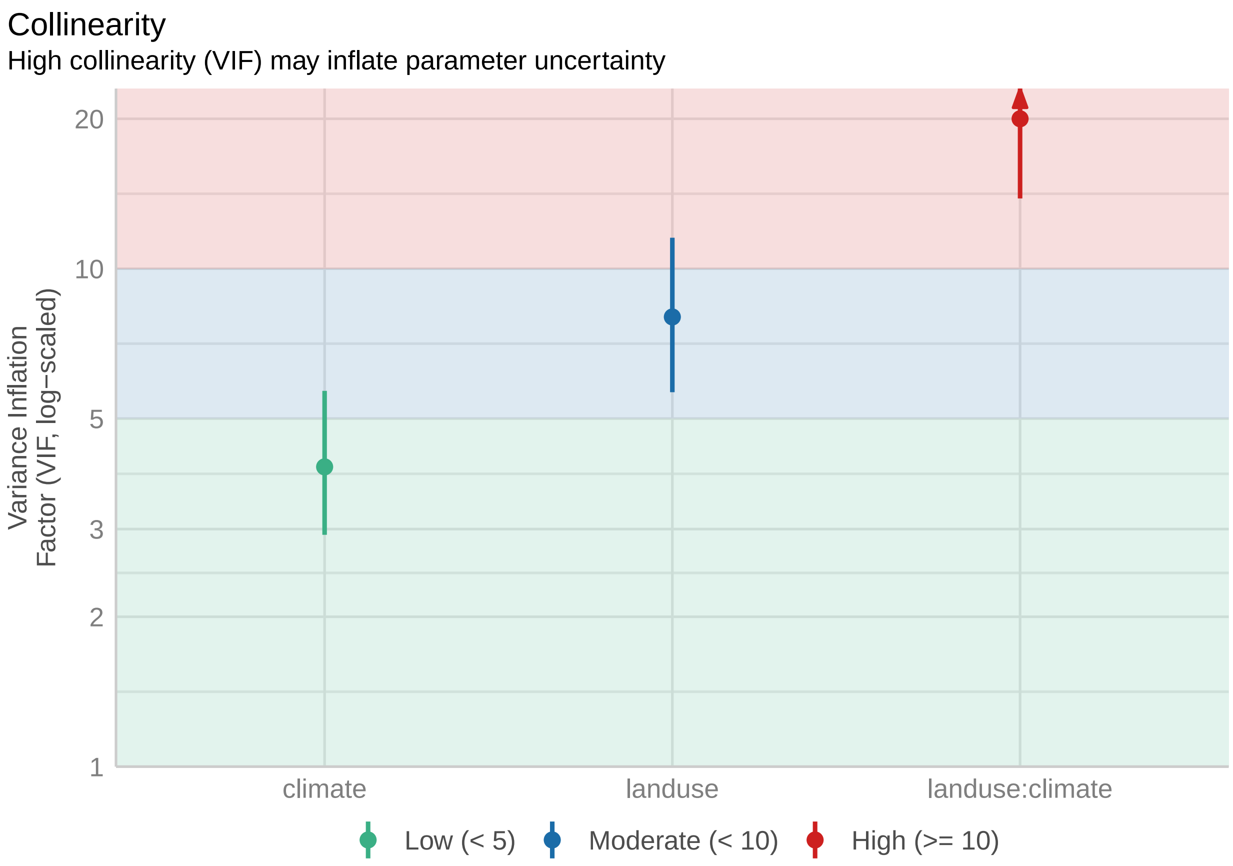
Collinearity
High collinearity (VIF) may inflate parameter uncertainty
Variance Inflation Factor (VIF, log−scaled)
20
10
5
3
2
1
climate
landuse
landuse:climate
Low (< 5)
Moderate (< 10)
High (>= 10)

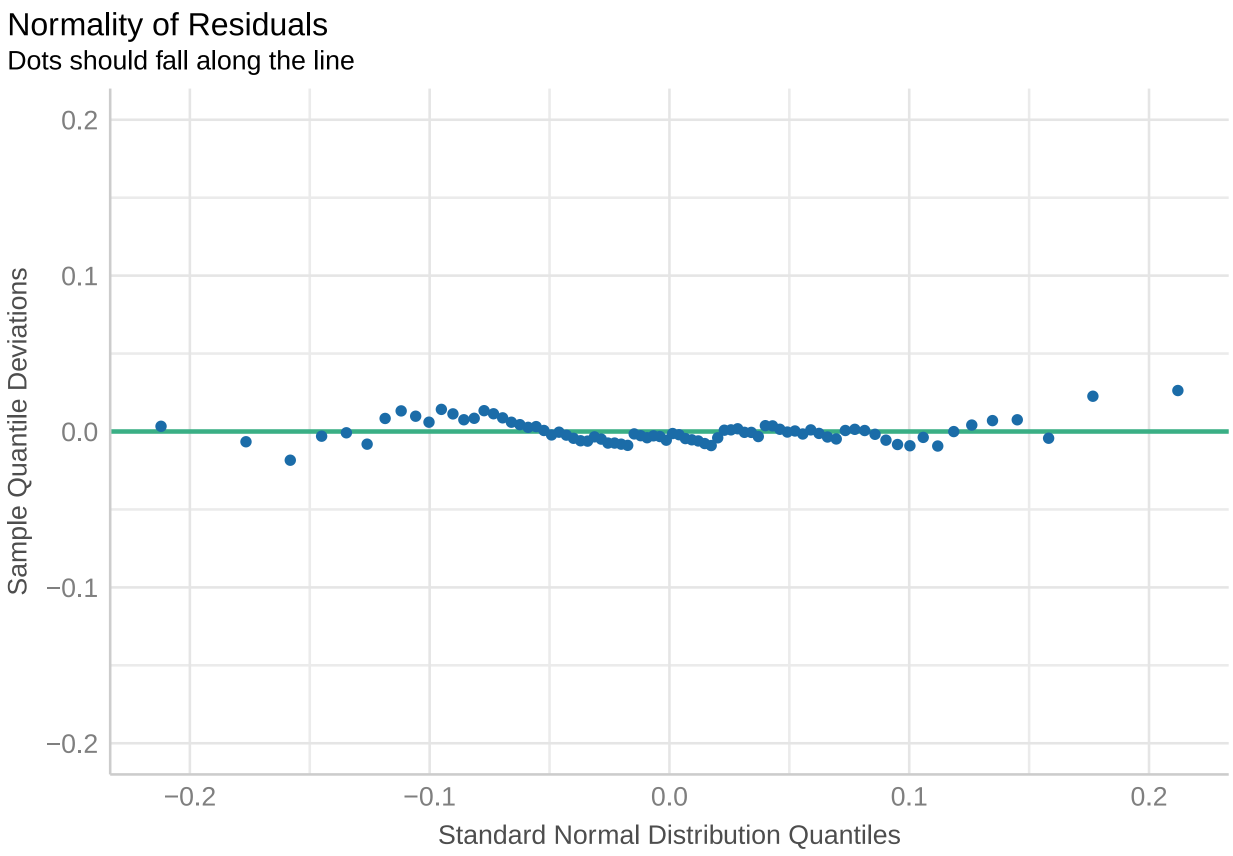
Normality of Residuals
Dots should fall along the line
Sample Quantile Deviations
0.2
0.1
0.0
−0.1
−0.2
−0.2
−0.1
0.0
0.1
0.2
Standard Normal Distribution Quantiles

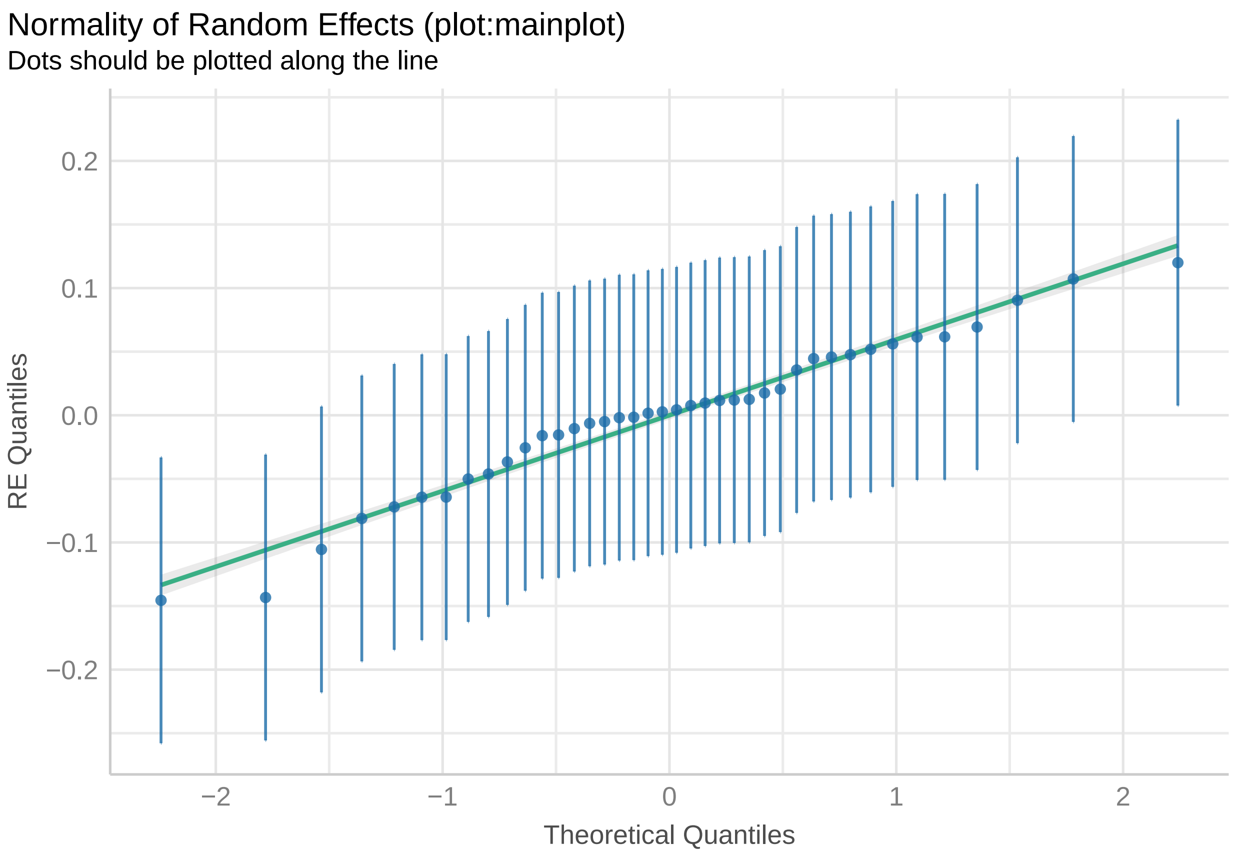
Normality of Random Effects (plot:mainplot)
Dots should be plotted along the line
0.2
0.1
0.0
−0.1
−0.2
RE Quantiles
−2
−1
0
1
2
Theoretical Quantiles

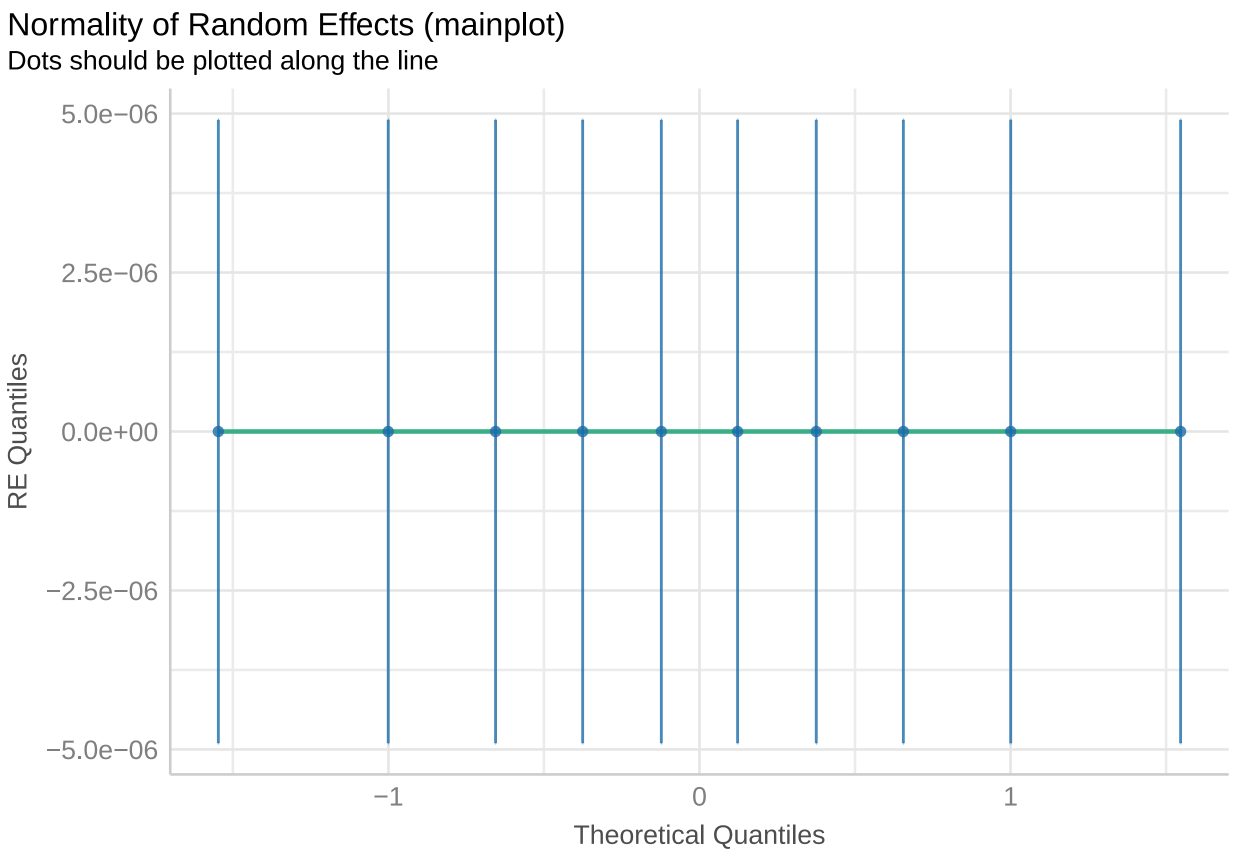
Normality of Random Effects (mainplot)
Dots should be plotted along the line
5.0e−06
2.5e−06
0.0e+00
−2.5e−06
−5.0e−06
RE Quantiles
−1
0
1
Theoretical Quantiles

Model outputs:

ANOVA type I:

```
## Type I Analysis of Variance Table with Kenward-Roger's method
##                  Sum Sq Mean Sq NumDF DenDF F value   Pr(>F)
## landuse         2.18911 0.72970     3    24 66.4110 9.12e-12 ***
## climate         0.07352 0.07352     1     8  6.6914  0.03227 *
## landuse:climate 0.04729 0.01576     3    24  1.4346  0.25732
## ---
## Signif. codes:  0 '***' 0.001 '**' 0.01 '*' 0.05 '.' 0.1 ' ' 1
```

Tukey test:

```
##
##   Simultaneous Tests for General Linear Hypotheses
##
## Multiple Comparisons of Means: Tukey Contrasts
##
##
## Fit: lmer(formula = "log(resp) ~ landuse * climate + (1|mainplot/plot) + (1|rep)",
##     data = filter(df, temperature == 20))
##
## Linear Hypotheses:
##               Estimate Std. Error z value Pr(>|z|)
## IG - EM == 0 -0.307992   0.072406  -4.254  < 0.001 ***
## OF - EM == 0 -0.572743   0.072406  -7.910  < 0.001 ***
## CF - EM == 0 -0.565282   0.072406  -7.807  < 0.001 ***
## OF - IG == 0 -0.264751   0.072406  -3.656  0.00149 **
## CF - IG == 0 -0.257290   0.072406  -3.553  0.00230 **
## CF - OF == 0  0.007461   0.072406   0.103  0.99961
## ---
## Signif. codes:  0 '***' 0.001 '**' 0.01 '*' 0.05 '.' 0.1 ' ' 1
## (Adjusted p values reported -- single-step method)
```

Supplementary Figure S2: log-transformed respiration

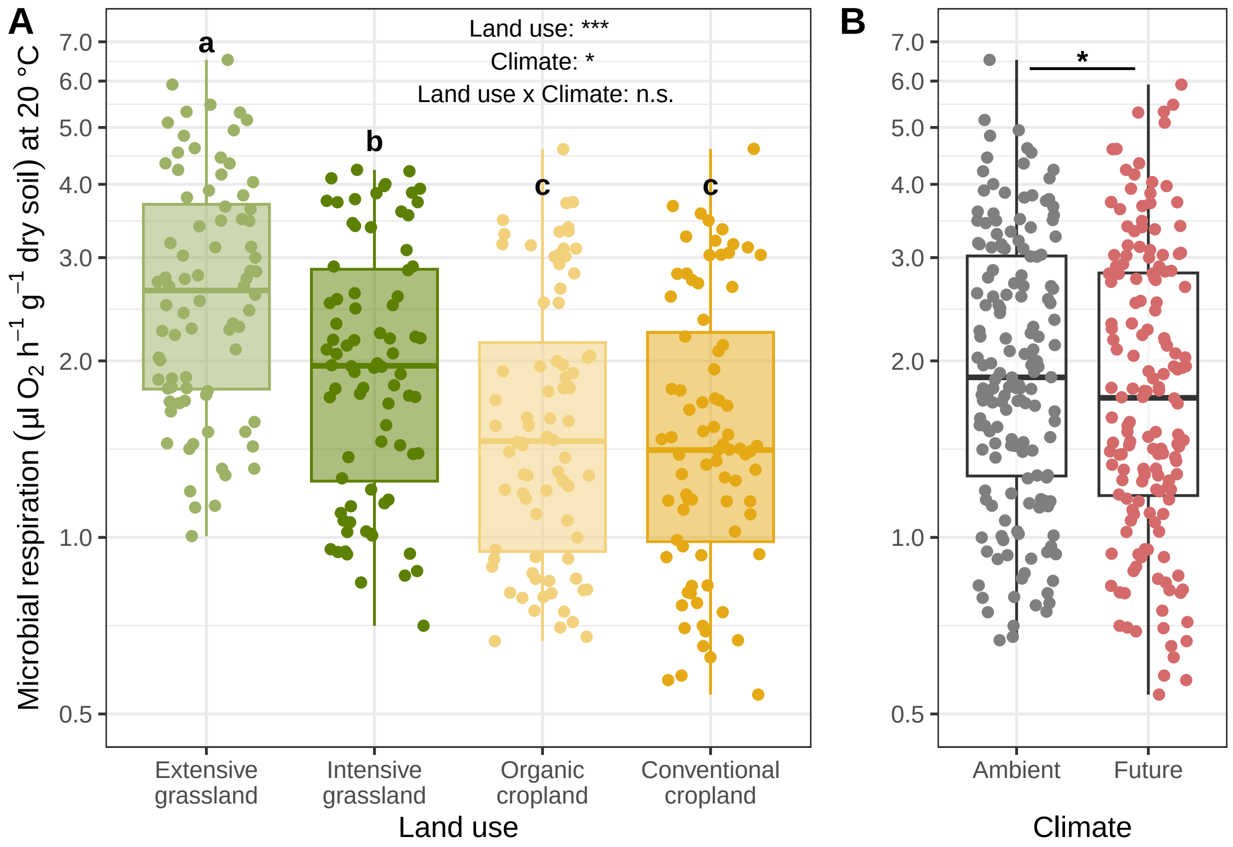

Figure 3: temperature treatment interaction with land use and climate

Supplementary material S2: statistical model fit and quality control

Statistical model:

```
model = lmer(
  formula = 'log(resp) ~ temperature * landuse * climate + (1|mainplot/plot) + (1|rep)',
            data = df)
```

Fit quality:

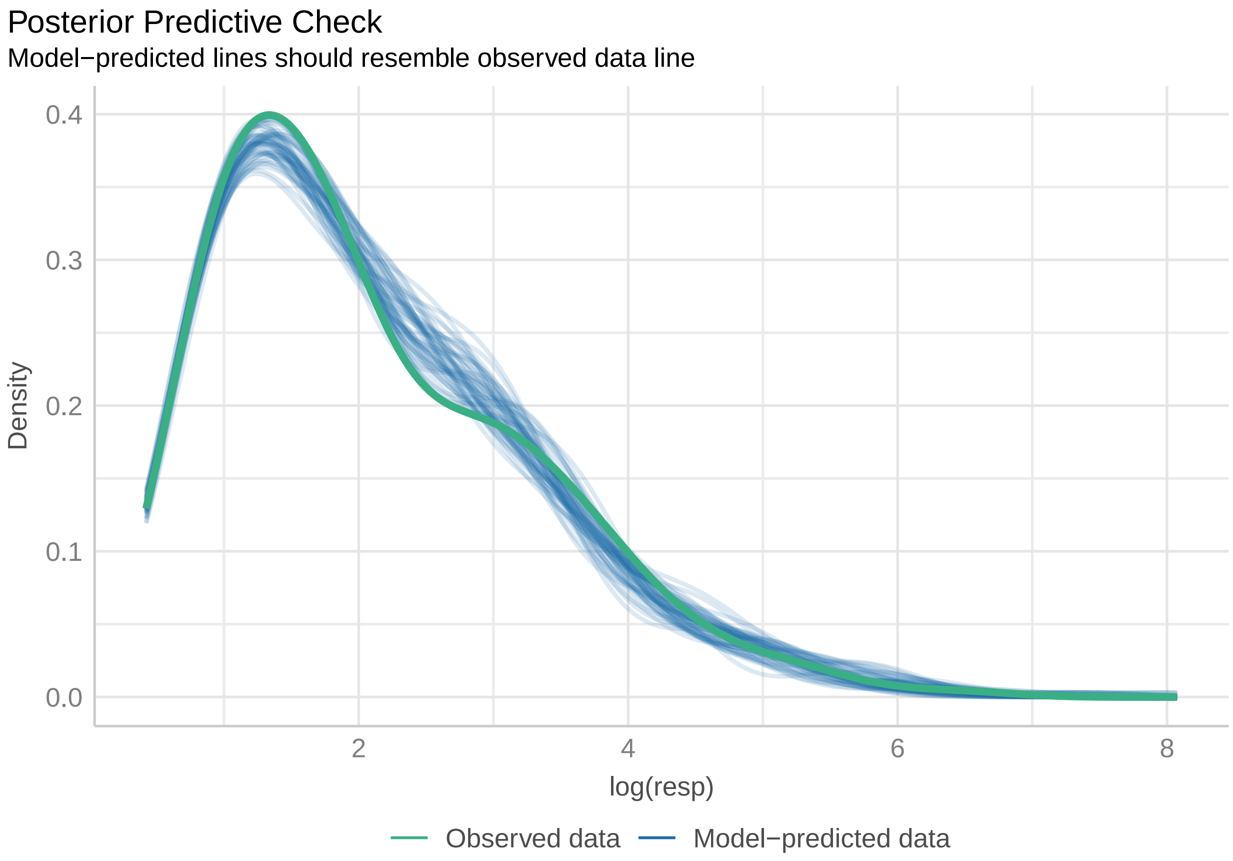

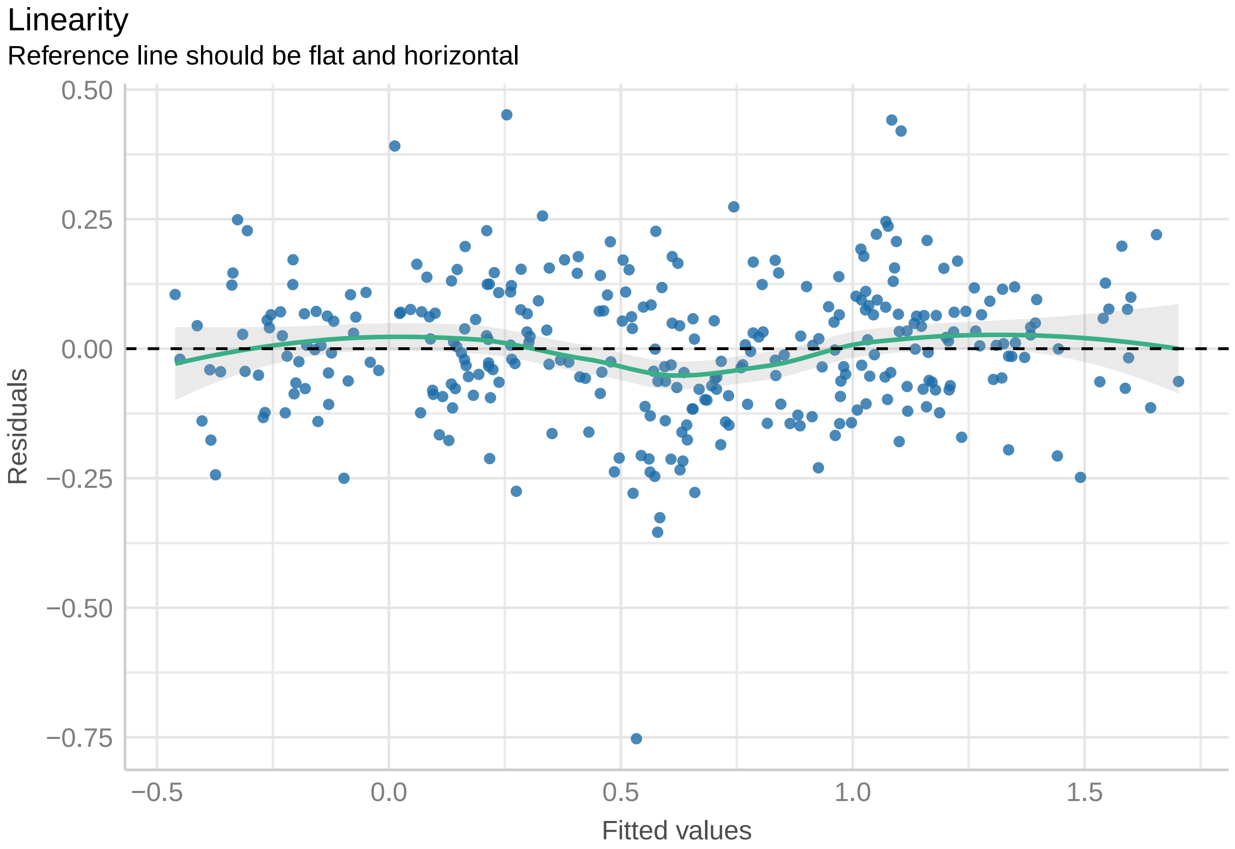
Linearity
Reference line should be flat and horizontal
0.50
0.25
0.00
−0.25
−0.50
−0.75
Residuals
−0.5
0.0
0.5
1.0
1.5
Fitted values

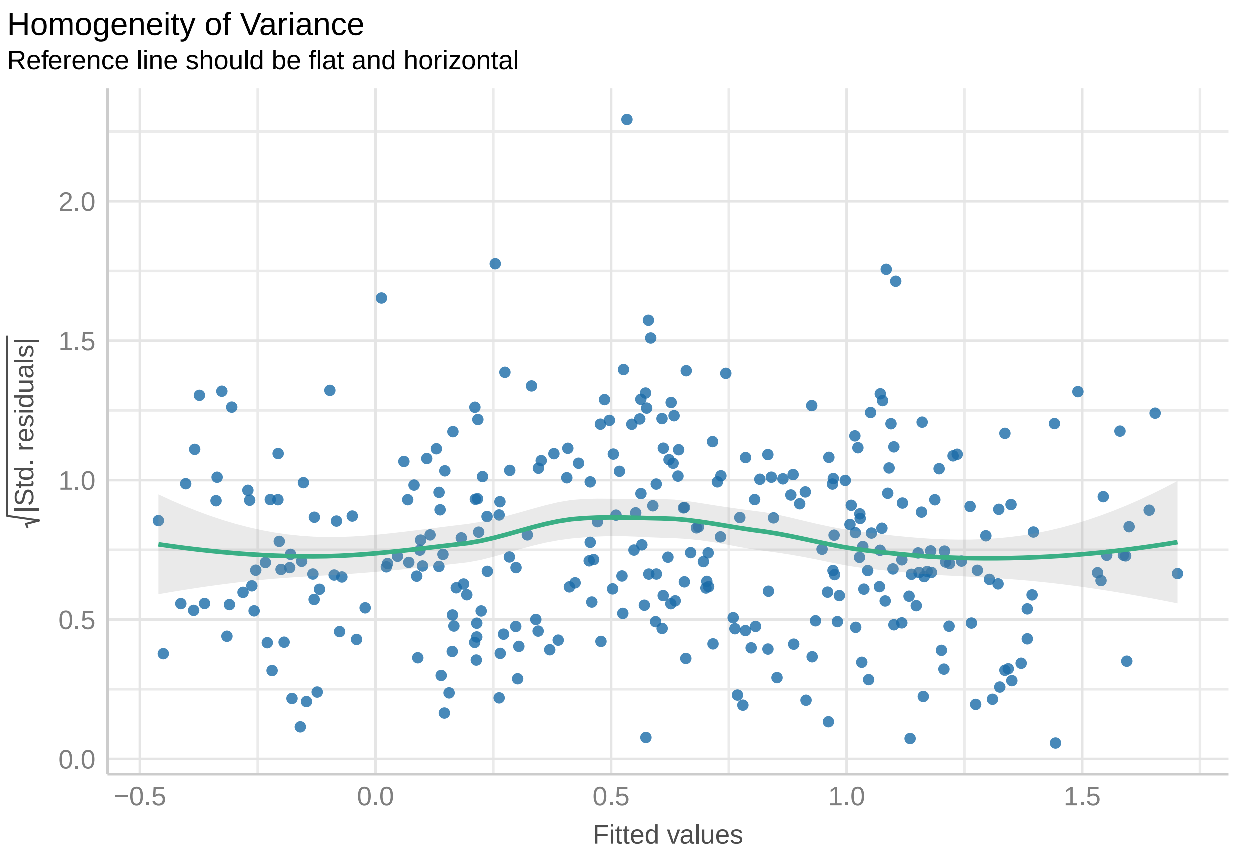
Homogeneity of Variance
Reference line should be flat and horizontal
√|Std. residuals|
2.0
1.5
1.0
0.5
0.0
−0.5
0.0
0.5
1.0
1.5
Fitted values

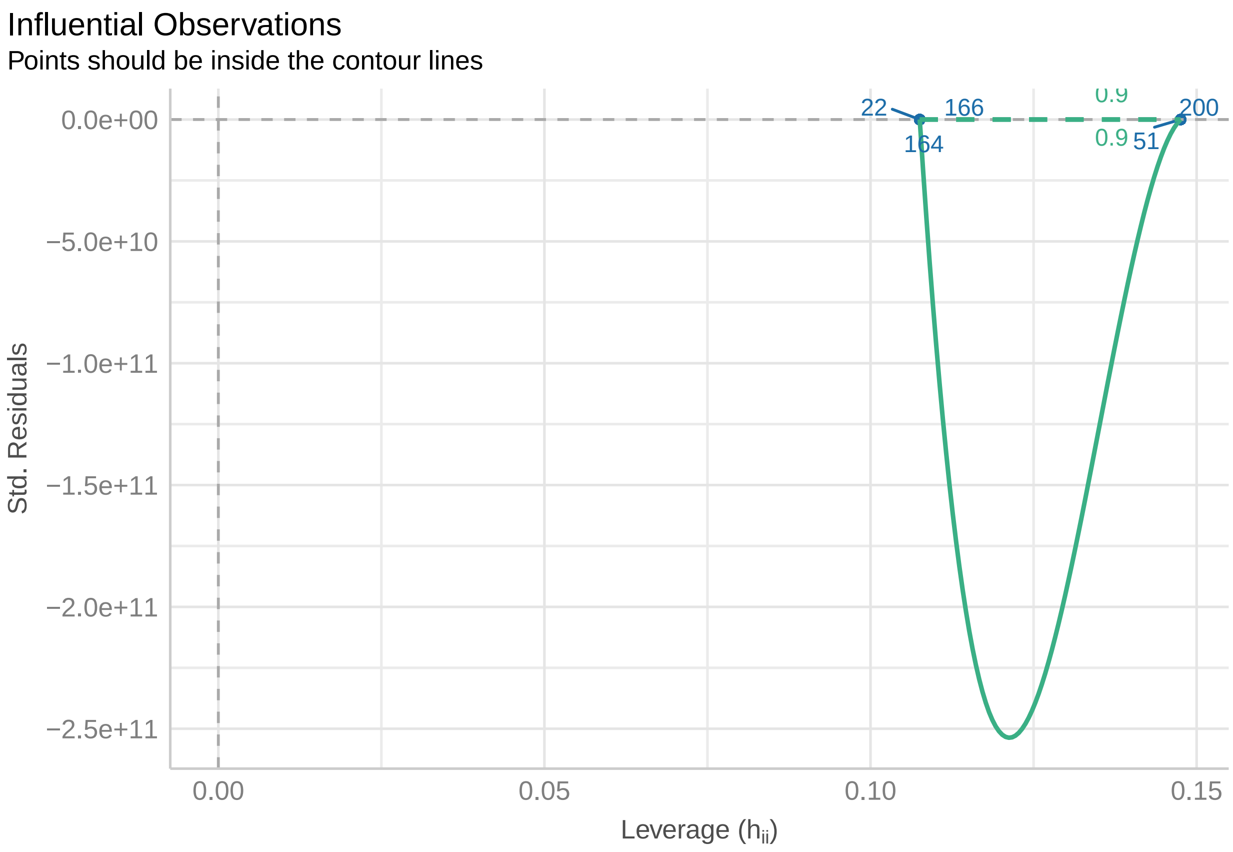
Influential Observations
Points should be inside the contour lines
Std. Residuals
0.0e+00
−5.0e+10
−1.0e+11
−1.5e+11
−2.0e+11
−2.5e+11
22
166
164
0.9
0.9
51
200
0.00
0.05
0.10
0.15
Leverage (h_{ii})

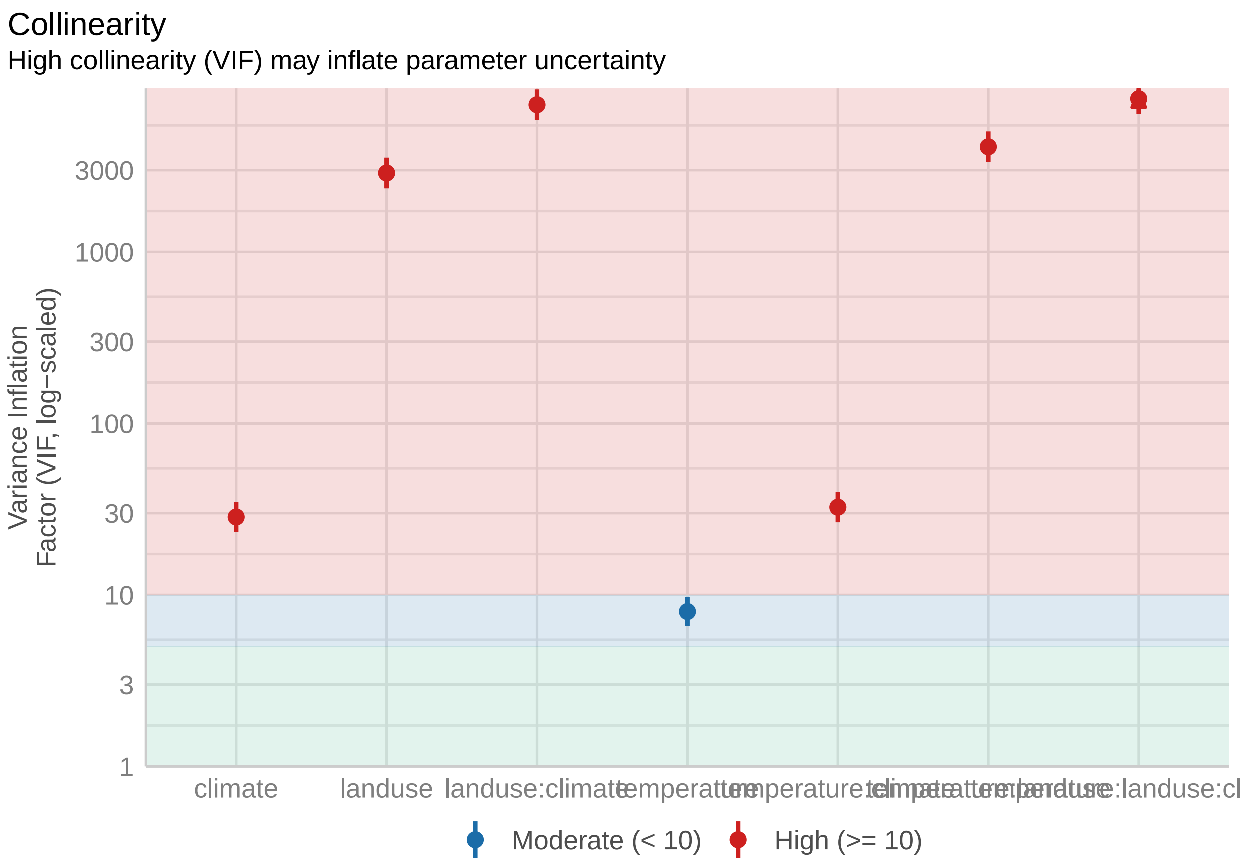

Collinearity
High collinearity (VIF) may inflate parameter uncertainty
Variance Inflation
Factor (VIF, log−scaled)
3000
1000
300
100
30
10
3
1
climate
landuse
landuse:climate
Moderate (< 10)
High (>= 10)

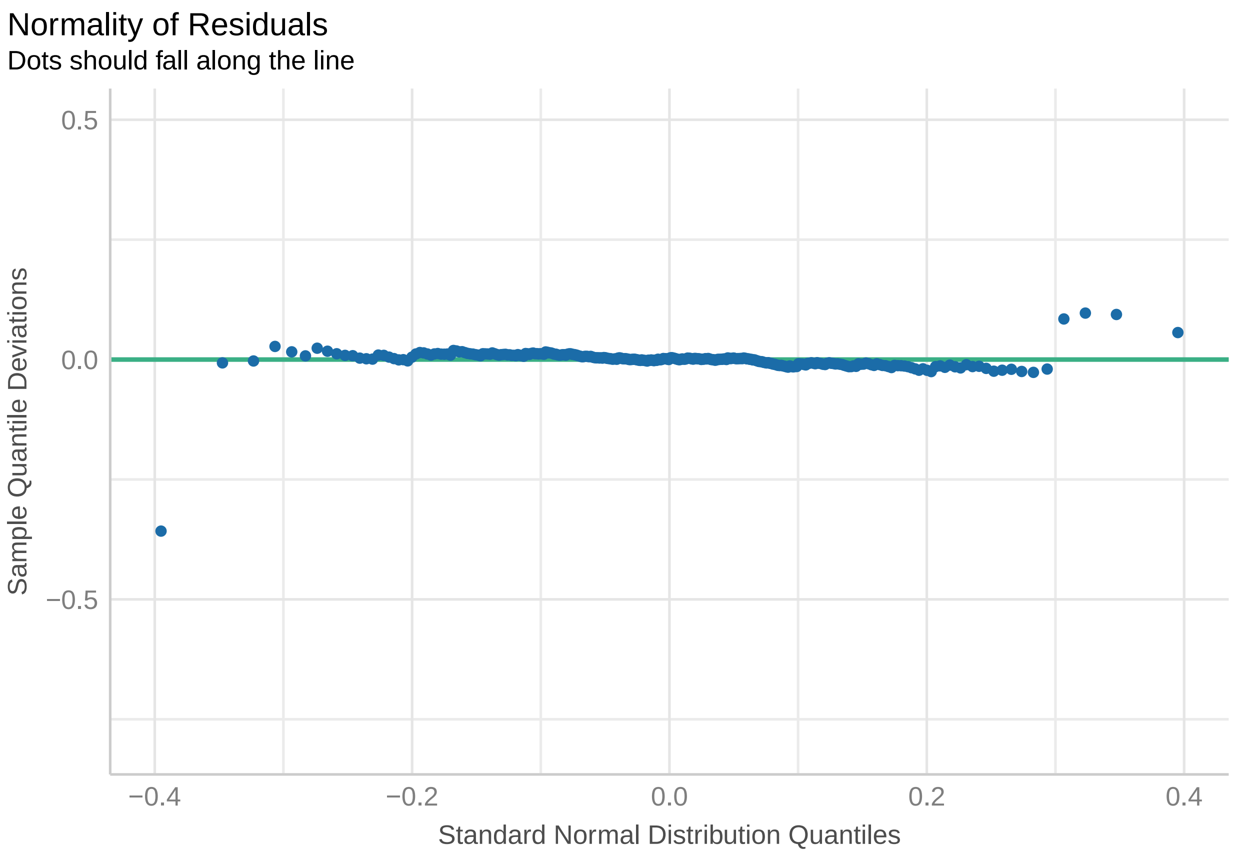
Normality of Residuals
Dots should fall along the line
Sample Quantile Deviations
0.5
0.0
−0.5
−0.4
−0.2
0.0
0.2
0.4
Standard Normal Distribution Quantiles

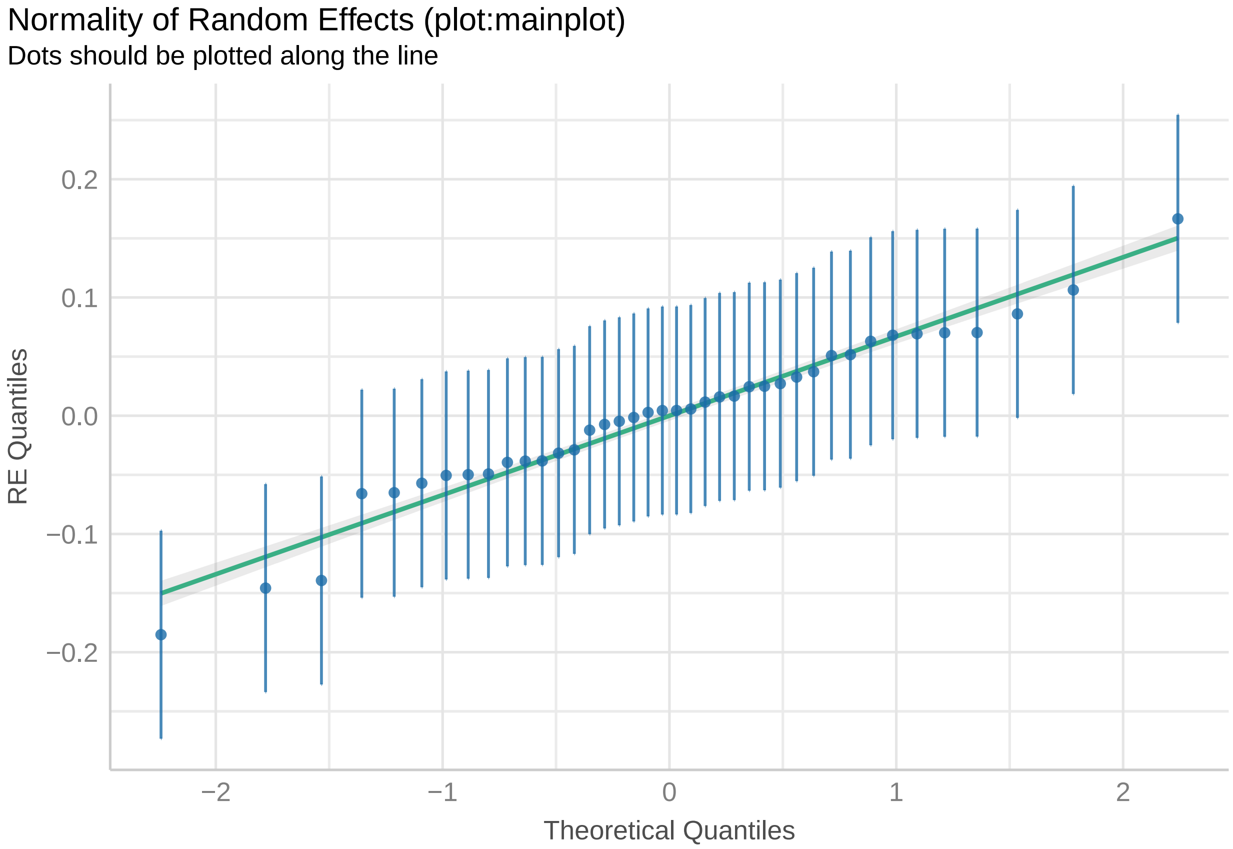
Normality of Random Effects (plot:mainplot)
Dots should be plotted along the line
0.2
0.1
0.0
−0.1
−0.2
RE Quantiles
−2
−1
0
1
2
Theoretical Quantiles

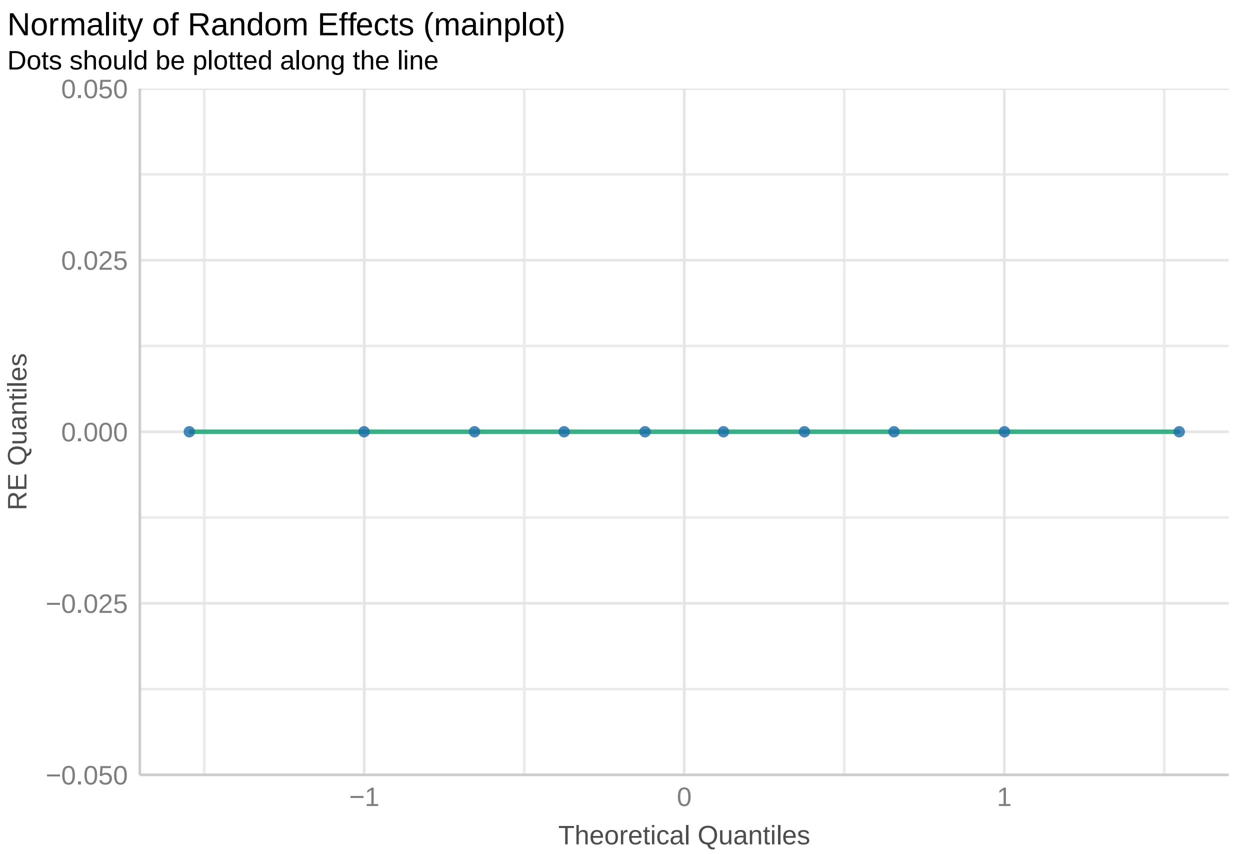
Normality of Random Effects (mainplot)
Dots should be plotted along the line
0.050
0.025
0.000
−0.025
−0.050
RE Quantiles
−1
0
1
Theoretical Quantiles

Model outputs:

ANOVA type I:

```
## Type I Analysis of Variance Table with Kenward-Roger's method
##                              Sum Sq Mean Sq NumDF DenDF   F value    Pr(>F)
## temperature                  70.336  70.336     1   271 3432.4299 < 2.2e-16 ***
## landuse                       4.027   1.342     3    24   65.5042 1.056e-11 ***
## climate                       0.156   0.156     1     8    7.6288  0.024598 *
## temperature:landuse           0.295   0.098     3   271    4.7980  0.002837 **
## temperature:climate           0.045   0.045     1   271    2.1972  0.139426
## landuse:climate               0.073   0.024     3    24    1.1813  0.337704
## temperature:landuse:climate  0.048   0.016     3   271    0.7791  0.506490
## ---
## Signif. codes:  0 '***' 0.001 '**' 0.01 '*' 0.05 '.' 0.1 ' ' 1
```

Supplementary Figure S3: log-transformed respiration

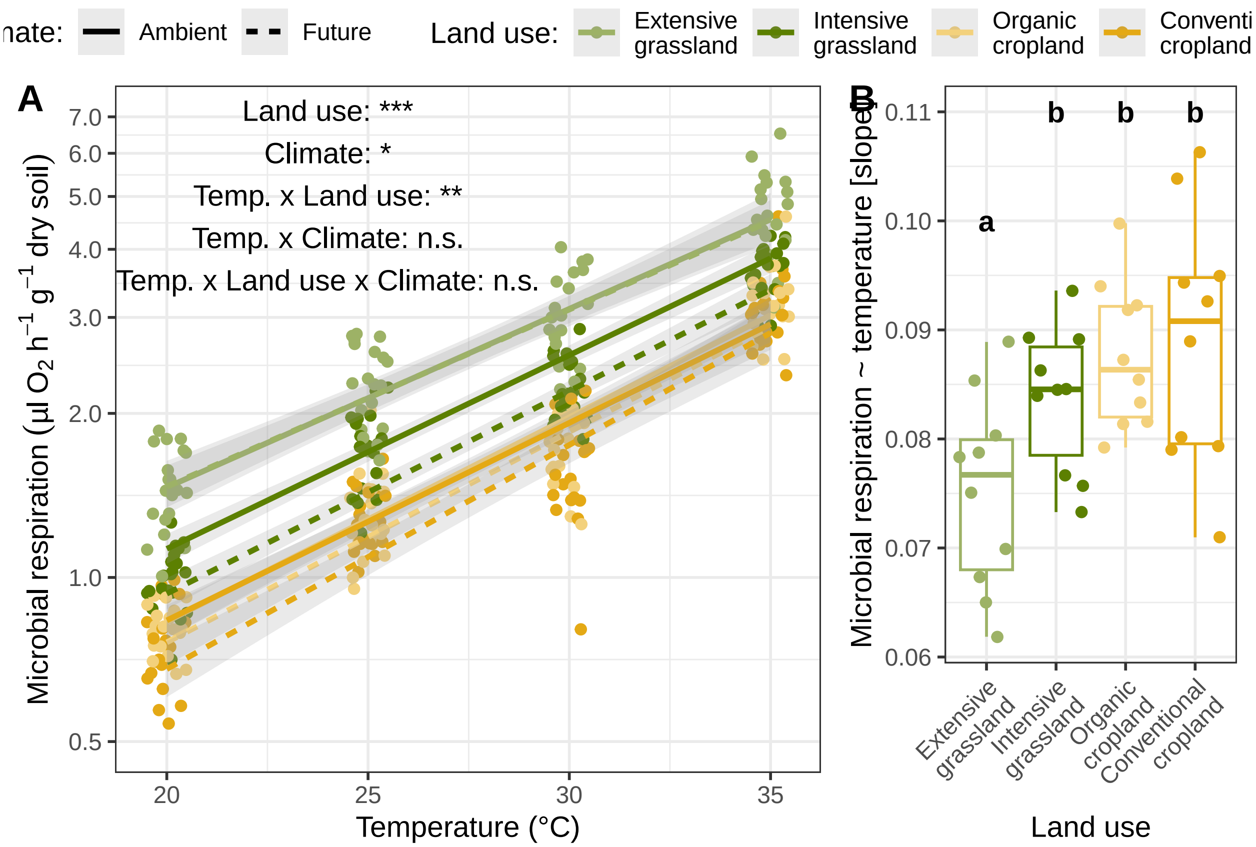

Figure 4

Supplementary material S3: land use and climate effects on soil microbial community

Soil microbial biomass

Model

```
mod.cmic = lmer(formula = 'cmic ~ (landuse * climate) + (1|mainplot) + (1|rep)',
     data = df.2)
```

Model quality

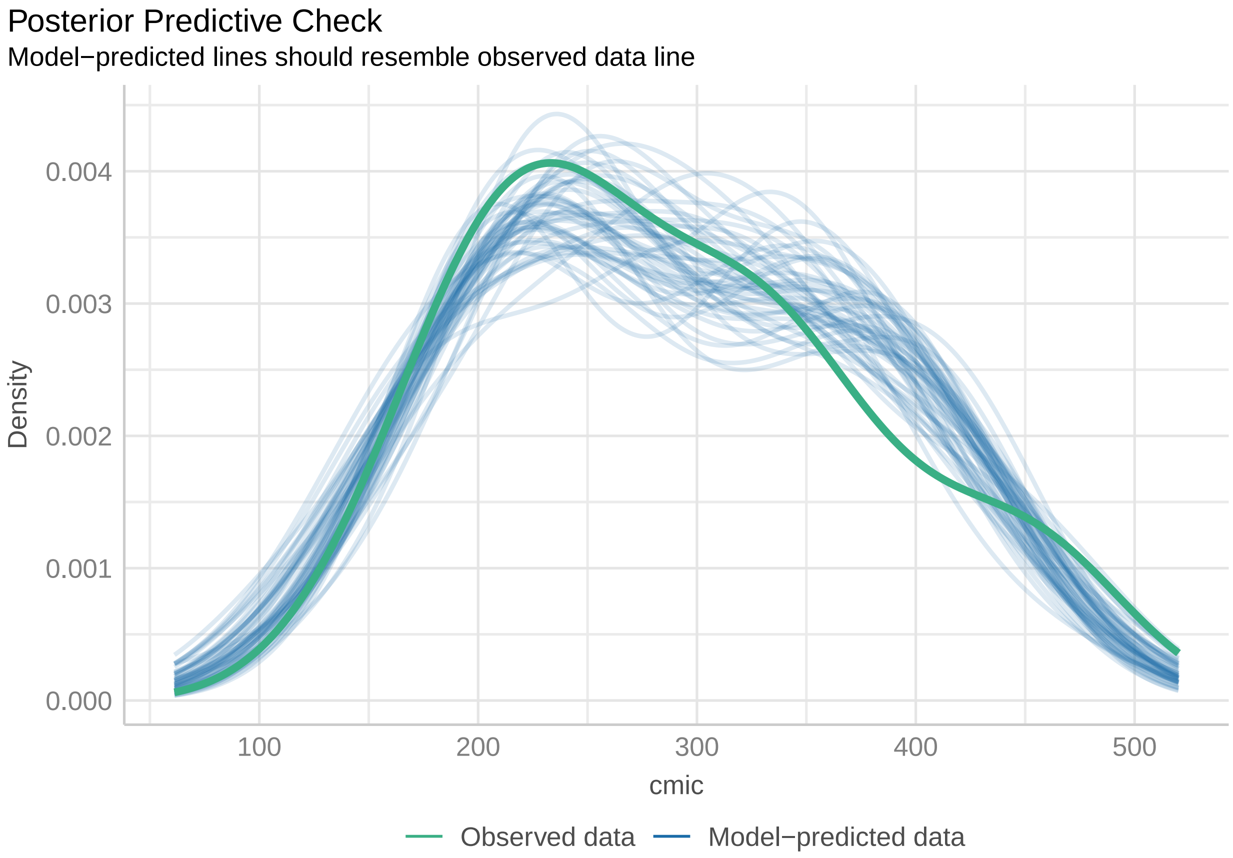

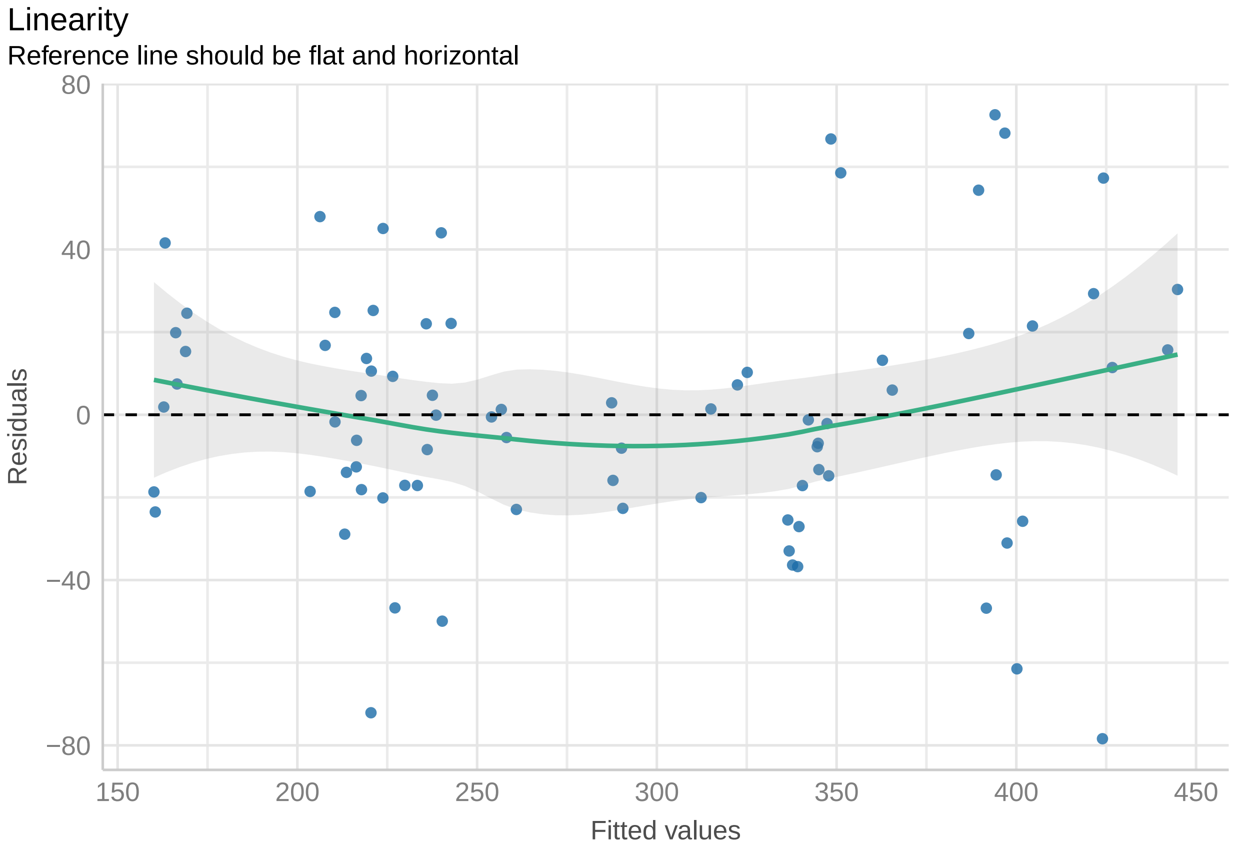
Linearity
Reference line should be flat and horizontal
Residuals
80
40
0
−40
−80
150
200
250
300
350
400
450
Fitted values

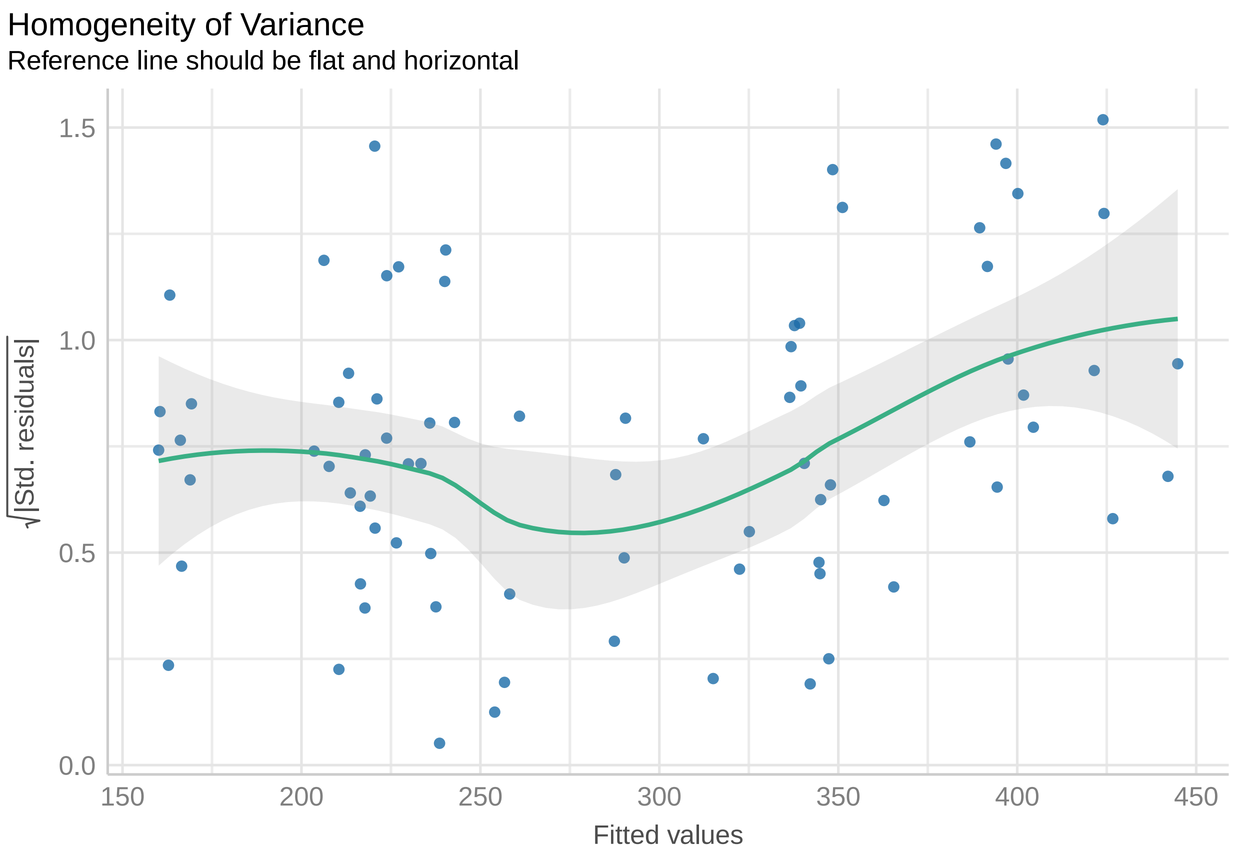
Homogeneity of Variance
Reference line should be flat and horizontal
√|Std. residuals|
1.5
1.0
0.5
0.0
150
200
250
300
350
400
450
Fitted values

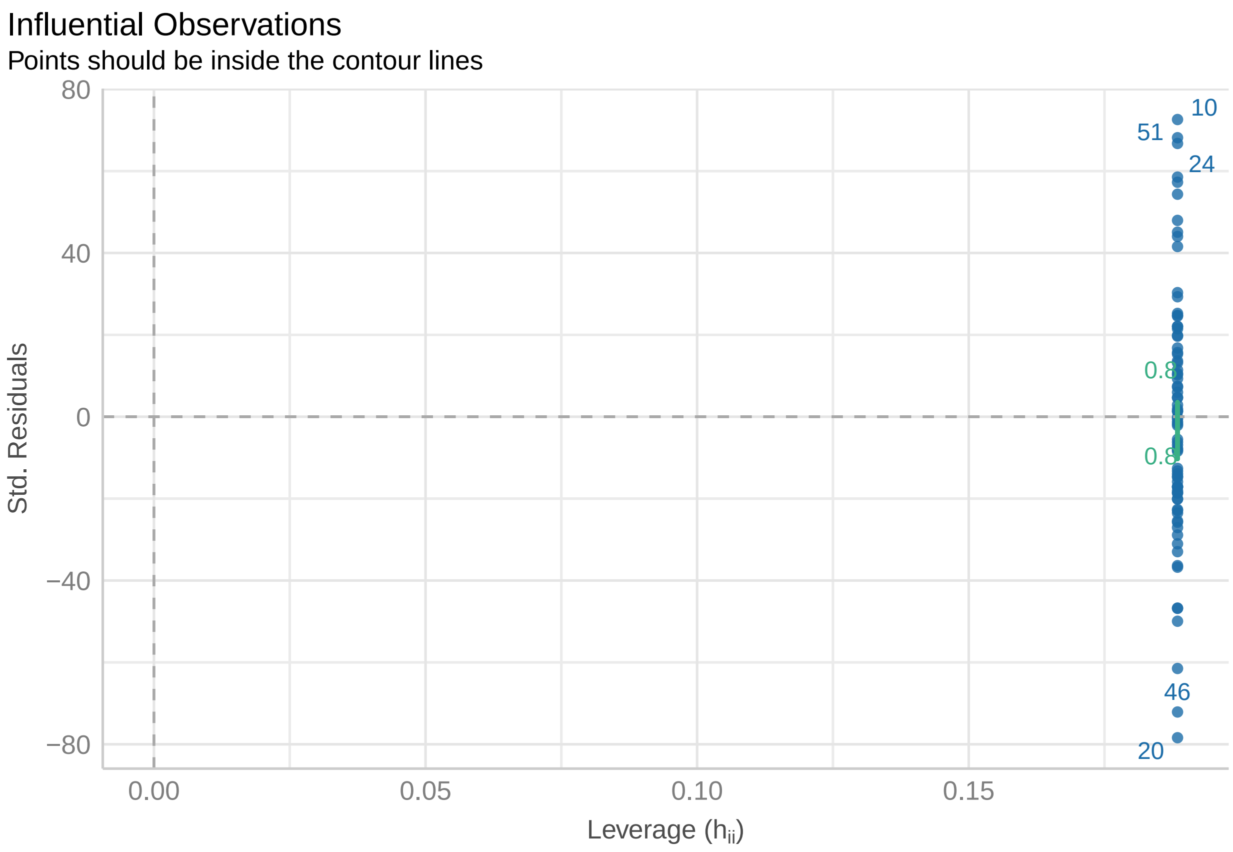

ANOVA

```
## Type I Analysis of Variance Table with Satterthwaite's method
##                  Sum Sq Mean Sq NumDF DenDF  F value Pr(>F)
## landuse          477008  159003     3    63 137.5168 <2e-16 ***
## climate            3128    3128     1     8   2.7055 0.1386
## landuse:climate    2342     781     3    63   0.6750 0.5706
## ---
## Signif. codes:  0 '***' 0.001 '**' 0.01 '*' 0.05 '.' 0.1 ' ' 1
```

Tukey test

```
##
##   Simultaneous Tests for General Linear Hypotheses
##
## Multiple Comparisons of Means: Tukey Contrasts
##
##
## Fit: lmer(formula = "cmic ~ (landuse * climate) + (1|mainplot) + (1|rep)",
##     data = df.2)
##
## Linear Hypotheses:
##               Estimate Std. Error z value Pr(>|z|)
## IG - EM == 0   -49.004     15.207  -3.222  0.00703 **
## OF - EM == 0 -176.334     15.207 -11.596  < 0.001 ***
## CF - EM == 0 -170.278     15.207 -11.197  < 0.001 ***
## OF - IG == 0 -127.330     15.207  -8.373  < 0.001 ***
## CF - IG == 0 -121.275     15.207  -7.975  < 0.001 ***
```

```
## CF - OF == 0    6.056     15.207   0.398  0.97862
## ---
## Signif. codes:  0 '***' 0.001 '**' 0.01 '*' 0.05 '.' 0.1 ' ' 1
## (Adjusted p values reported -- single-step method)
```

Total fungal biomass

Model

```
mod.fungi = lmer(formula = 'log(all_fungi) ~ (landuse * climate) + (1|mainplot)',
     data = df.plfa)
```

Model quality

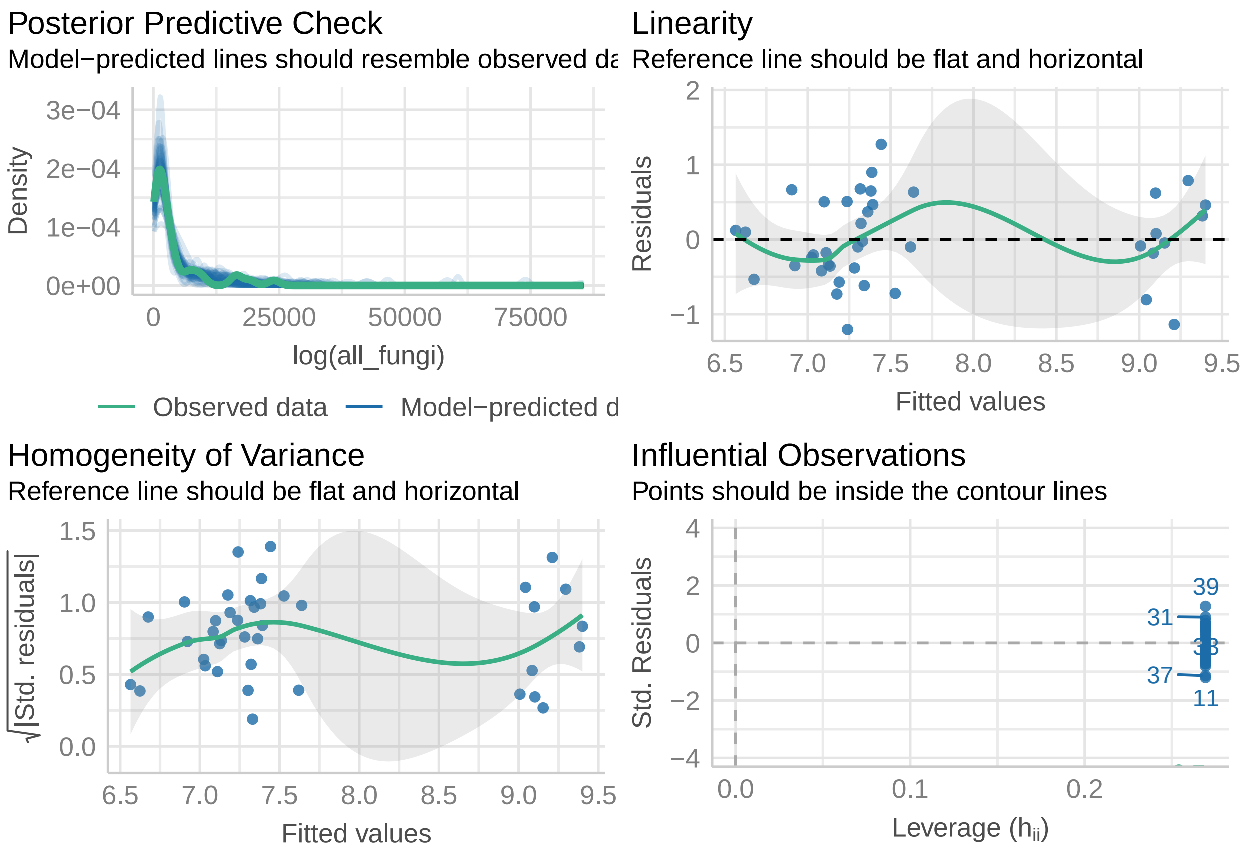

ANOVA

```
## Type I Analysis of Variance Table with Satterthwaite's method
##                  Sum Sq Mean Sq NumDF DenDF F value    Pr(>F)
## landuse         30.4710 10.1570     3    24 23.3383 2.698e-07 ***
## climate          0.0372  0.0372     1     8  0.0854    0.7776
## landuse:climate  0.5840  0.1947     3    24  0.4473    0.7215
## ---
## Signif. codes:  0 '***' 0.001 '**' 0.01 '*' 0.05 '.' 0.1 ' ' 1
```

Tukey test

```
##
##   Simultaneous Tests for General Linear Hypotheses
##
## Multiple Comparisons of Means: Tukey Contrasts
##
##
## Fit: lmer(formula = "log(all_fungi) ~ (landuse * climate) + (1|mainplot)",
##     data = df.plfa)
```

```
##
## Linear Hypotheses:
##              Estimate Std. Error z value Pr(>|z|)
## IG - EM == 0 -1.76805    0.41723  -4.238 0.000121 ***
## OF - EM == 0 -1.97422    0.41723  -4.732  < 1e-04 ***
## CF - EM == 0 -1.90908    0.41723  -4.576  < 1e-04 ***
## OF - IG == 0 -0.20617    0.41723  -0.494 0.960419
## CF - IG == 0 -0.14103    0.41723  -0.338 0.986708
## CF - OF == 0  0.06514    0.41723   0.156 0.998646
## ---
## Signif. codes:  0 '***' 0.001 '**' 0.01 '*' 0.05 '.' 0.1 ' ' 1
## (Adjusted p values reported -- single-step method)
```

Total bacterial biomass

Model

```
mod.bacteria = lmer(formula = 'log(all_bacteria) ~ (landuse * climate) + (1|mainplot)',
     data = df.plfa)
```

Model quality

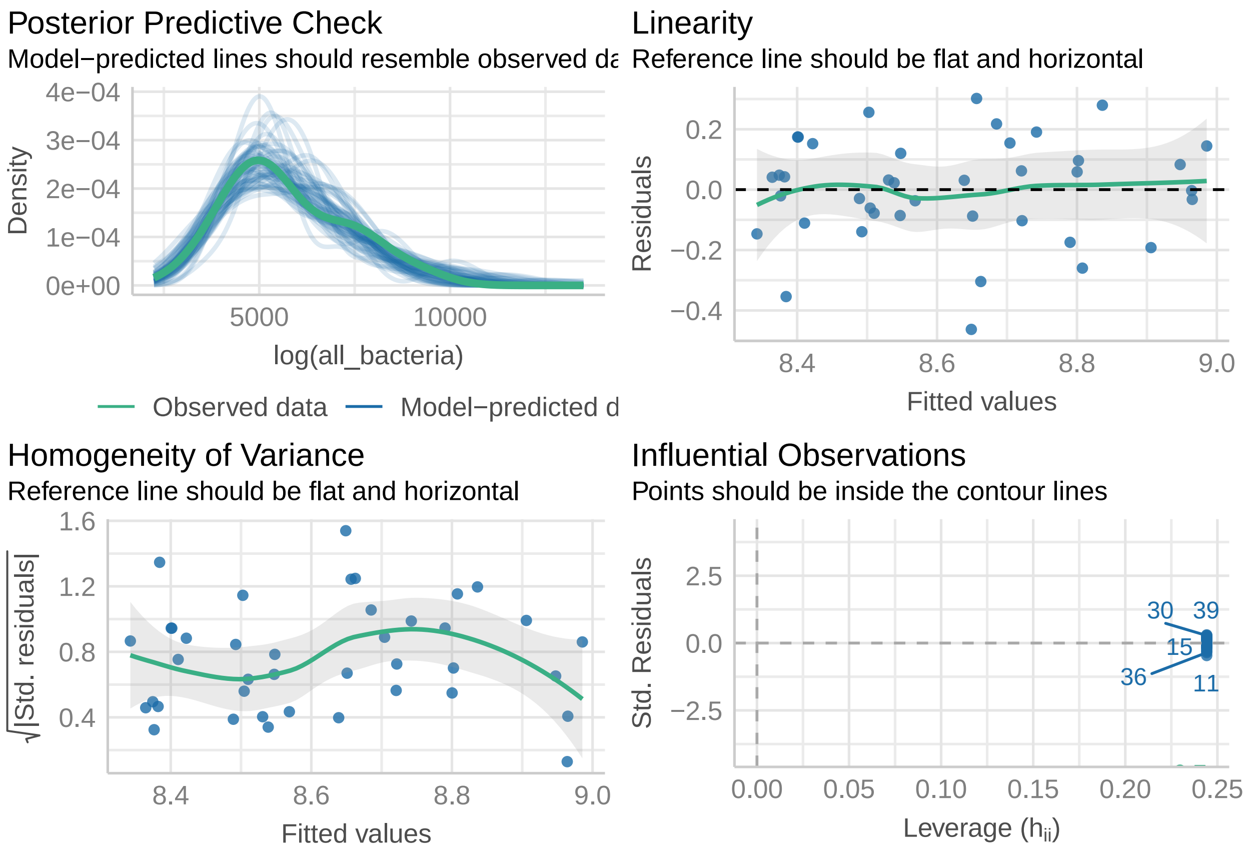

ANOVA

```
## Type I Analysis of Variance Table with Satterthwaite's method
##                   Sum Sq Mean Sq NumDF DenDF F value    Pr(>F)
## landuse          1.35965 0.45322     3    24 11.8891 5.729e-05 ***
## climate          0.02715 0.02715     1     8  0.7121    0.4233
## landuse:climate 0.02794 0.00931     3    24  0.2443    0.8646
## ---
## Signif. codes:  0 '***' 0.001 '**' 0.01 '*' 0.05 '.' 0.1 ' ' 1
```

Tukey test

```
##
##   Simultaneous Tests for General Linear Hypotheses
##
## Multiple Comparisons of Means: Tukey Contrasts
##
##
## Fit: lmer(formula = "log(all_bacteria) ~ (landuse * climate) + (1|mainplot)",
##     data = df.plfa)
```

```
##
## Linear Hypotheses:
##              Estimate Std. Error z value Pr(>|z|)
## IG - EM == 0  -0.1513     0.1235  -1.225  0.61086
## OF - EM == 0  -0.4258     0.1235  -3.448  0.00334 **
## CF - EM == 0  -0.2977     0.1235  -2.411  0.07514 .
## OF - IG == 0  -0.2745     0.1235  -2.223  0.11722
## CF - IG == 0  -0.1464     0.1235  -1.186  0.63583
## CF - OF == 0   0.1281     0.1235   1.037  0.72766
## ---
## Signif. codes:  0 '***' 0.001 '**' 0.01 '*' 0.05 '.' 0.1 ' ' 1
## (Adjusted p values reported -- single-step method)
```

Fungal-to-bacterial ratio

Model

```
mod.bacteria = lmer(formula = 'log(f_b) ~ (landuse * climate) + (1|mainplot)',
     data = df.plfa)
```

Model quality

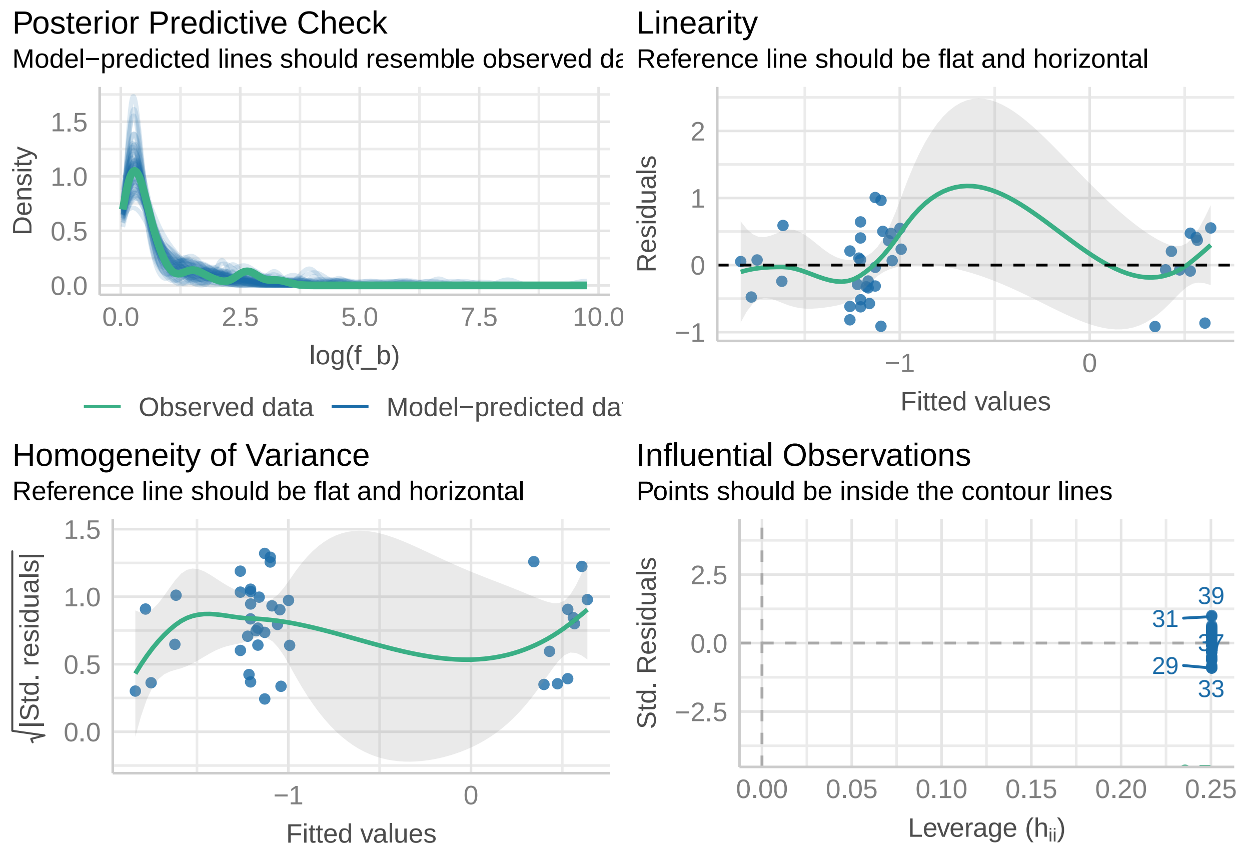

ANOVA

```
## Type I Analysis of Variance Table with Satterthwaite's method
##                   Sum Sq Mean Sq NumDF DenDF F value    Pr(>F)
## landuse          23.6860  7.8953     3    24 23.6619 2.389e-07 ***
## climate           0.1465  0.1465     1     8  0.4391    0.5262
## landuse:climate   0.5653  0.1884     3    24  0.5647    0.6436
## ---
## Signif. codes:  0 '***' 0.001 '**' 0.01 '*' 0.05 '.' 0.1 ' ' 1
```

Tukey test

```
##
##   Simultaneous Tests for General Linear Hypotheses
##
## Multiple Comparisons of Means: Tukey Contrasts
##
##
## Fit: lmer(formula = "log(f_b) ~ (landuse * climate) + (1|mainplot)",
##     data = df.plfa)
```

```
##
## Linear Hypotheses:
##                Estimate Std. Error z value Pr(>|z|)
## IG - EM == 0 -1.7363508  0.3653344  -4.753  < 1e-05 ***
## OF - EM == 0 -1.6962412  0.3653344  -4.643 1.51e-05 ***
## CF - EM == 0 -1.7359461  0.3653344  -4.752 1.34e-05 ***
## OF - IG == 0  0.0401096  0.3653344   0.110        1
## CF - IG == 0  0.0004048  0.3653344   0.001        1
## CF - OF == 0 -0.0397048  0.3653344  -0.109        1
## ---
## Signif. codes:  0 '***' 0.001 '**' 0.01 '*' 0.05 '.' 0.1 ' ' 1
## (Adjusted p values reported -- single-step method)
```

Supplementary material S4: soil microbial community effects on response to temperature treatment

Microbial biomass

Model

```r
lmer(formula = 'estimate ~ cmic + (1|mainplot) + (1|rep)',
     data = df.2)
```

Model quality

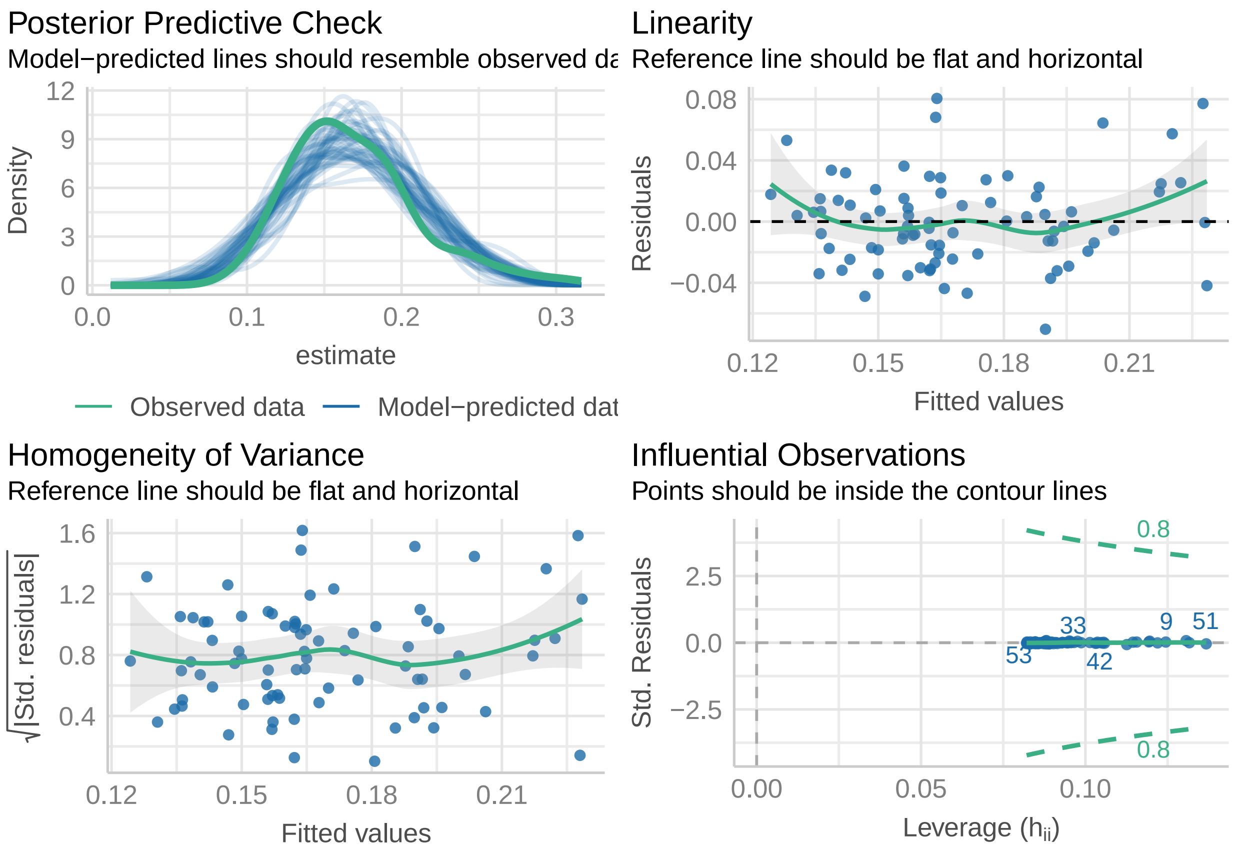

Summary

```r
summary(mod)
```

```
## Linear mixed model fit by REML. t-tests use Satterthwaite's method [
## lmerModLmerTest]
## Formula: "estimate ~ cmic + (1|mainplot) + (1|rep)"
##    Data: df.2
##
## REML criterion at convergence: -295.5
##
## Scaled residuals:
##      Min       1Q   Median       3Q      Max
## -2.28696 -0.64196 -0.05869  0.54163  2.61592
##
## Random effects:
##  Groups   Name        Variance  Std.Dev.
```

```
##  mainplot (Intercept) 0.0001906 0.01381
##  rep      (Intercept) 0.0000000 0.00000
##  Residual             0.0009460 0.03076
## Number of obs: 80, groups:  mainplot, 10; rep, 2
##
## Fixed effects:
##              Estimate Std. Error        df t value Pr(>|t|)
## (Intercept) 9.131e-02  1.287e-02 6.910e+01   7.092 8.99e-10 ***
## cmic        2.697e-04  4.022e-05 7.519e+01   6.704 3.28e-09 ***
## ---
## Signif. codes:  0 '***' 0.001 '**' 0.01 '*' 0.05 '.' 0.1 ' ' 1
##
## Correlation of Fixed Effects:
##      (Intr)
## cmic -0.902
## optimizer (nloptwrap) convergence code: 0 (OK)
## boundary (singular) fit: see help('isSingular')
```

Total fungal biomass

Model

```
lmer(formula = 'estimate ~ all_fungi + (1|mainplot)',
     data = df.1)
```

Model quality

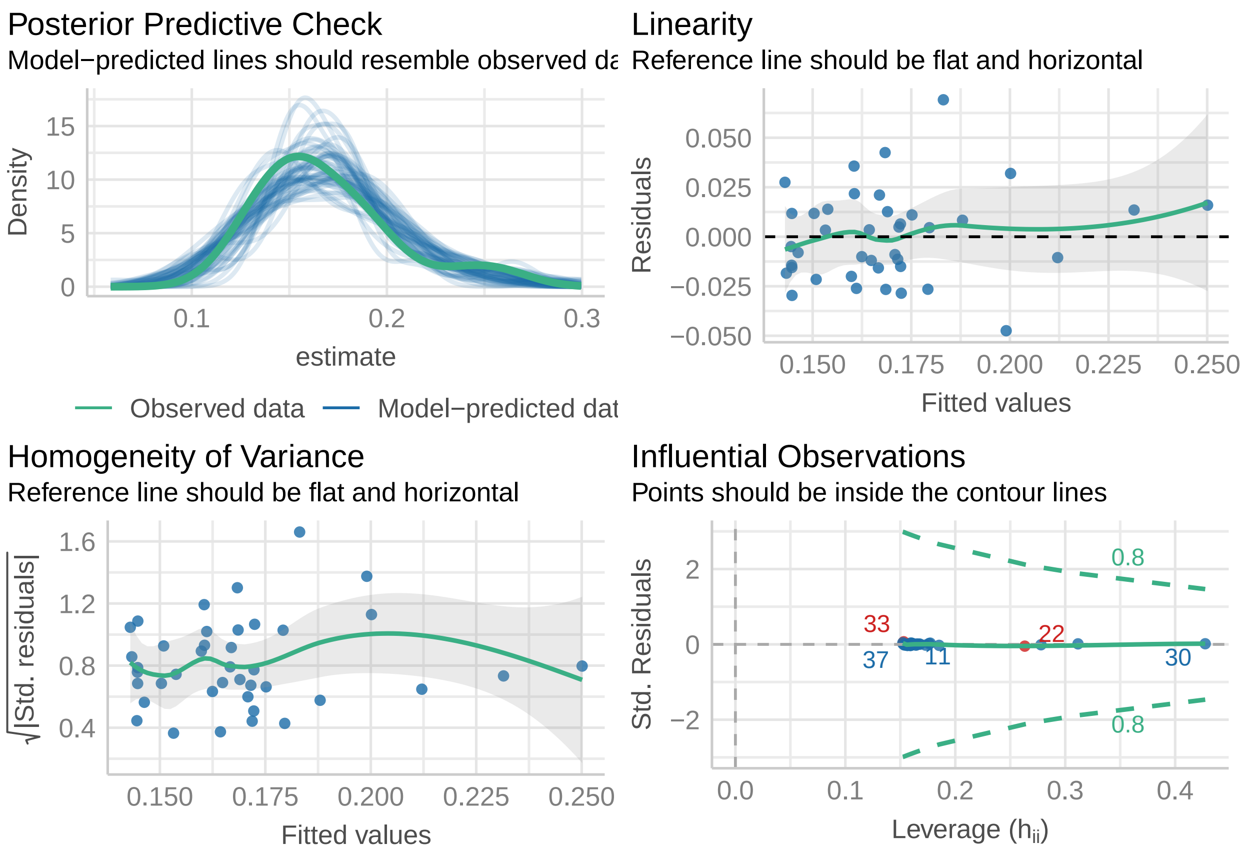

Summary

```
summary(mod)
```

```
## Linear mixed model fit by REML. t-tests use Satterthwaite's method [
## lmerModLmerTest]
## Formula: "estimate ~ all_fungi + (1|mainplot)"
##    Data: df.1
##
## REML criterion at convergence: -140.2
##
## Scaled residuals:
##      Min       1Q   Median       3Q      Max
## -1.89135 -0.62145 -0.03254  0.51293  2.75684
##
## Random effects:
##  Groups   Name        Variance  Std.Dev.
##  mainplot (Intercept) 0.0002045 0.01430
##  Residual             0.0006295 0.02509
## Number of obs: 40, groups:  mainplot, 10
```

```
##
## Fixed effects:
##              Estimate Std. Error        df t value Pr(>|t|)
## (Intercept) 1.530e-01  6.761e-03 1.365e+01  22.621 3.23e-12 ***
## all_fungi   3.887e-06  7.400e-07 3.202e+01   5.253 9.51e-06 ***
## ---
## Signif. codes:  0 '***' 0.001 '**' 0.01 '*' 0.05 '.' 0.1 ' ' 1
##
## Correlation of Fixed Effects:
##           (Intr)
## all_fungi -0.457
## fit warnings:
## Some predictor variables are on very different scales: consider rescaling
```

Total fungal biomass

Model

```r
lmer(formula = 'estimate ~ all_bacteria + (1|mainplot)',
     data = df.1)
```

Model quality

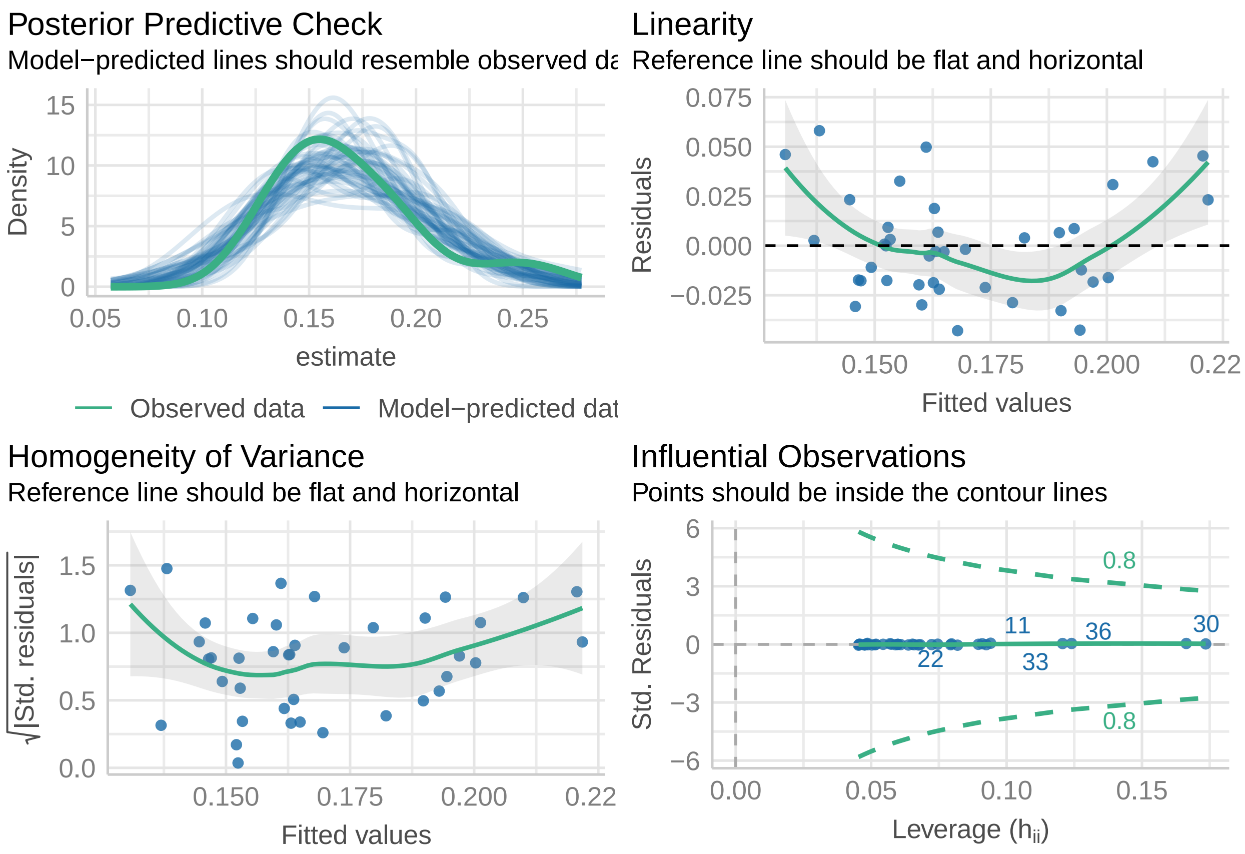

Summary

```r
summary(mod)
```

```
## Linear mixed model fit by REML. t-tests use Satterthwaite's method [
## lmerModLmerTest]
## Formula: "estimate ~ all_bacteria + (1|mainplot)"
##    Data: df.1
##
## REML criterion at convergence: -144.8
##
## Scaled residuals:
##      Min       1Q   Median       3Q      Max
## -1.60950 -0.69023 -0.08873  0.43728  2.17840
##
## Random effects:
##  Groups   Name        Variance  Std.Dev.
##  mainplot (Intercept) 1.764e-05 0.00420
##  Residual             7.109e-04 0.02666
## Number of obs: 40, groups:  mainplot, 10
```

```
##
## Fixed effects:
##               Estimate Std. Error        df t value Pr(>|t|)    
## (Intercept)  8.337e-02  1.663e-02 3.791e+01   5.012 1.29e-05 ***
## all_bacteria 1.499e-05  2.802e-06 3.668e+01   5.350 4.87e-06 ***
## ---
## Signif. codes:  0 '***' 0.001 '**' 0.01 '*' 0.05 '.' 0.1 ' ' 1
##
## Correlation of Fixed Effects:
##             (Intr)
## all_bacteri -0.964
## fit warnings:
## Some predictor variables are on very different scales: consider rescaling
```

Fungal-to-bacterial ratio

Model

```
lmer(formula = 'estimate ~ f_b + (1|mainplot)',
     data = df.1)
```

Model quality

```
mod = lmer(formula = 'estimate ~ f_b + (1|mainplot)',
     data = df.1)

a = plot(check_model(mod))
```

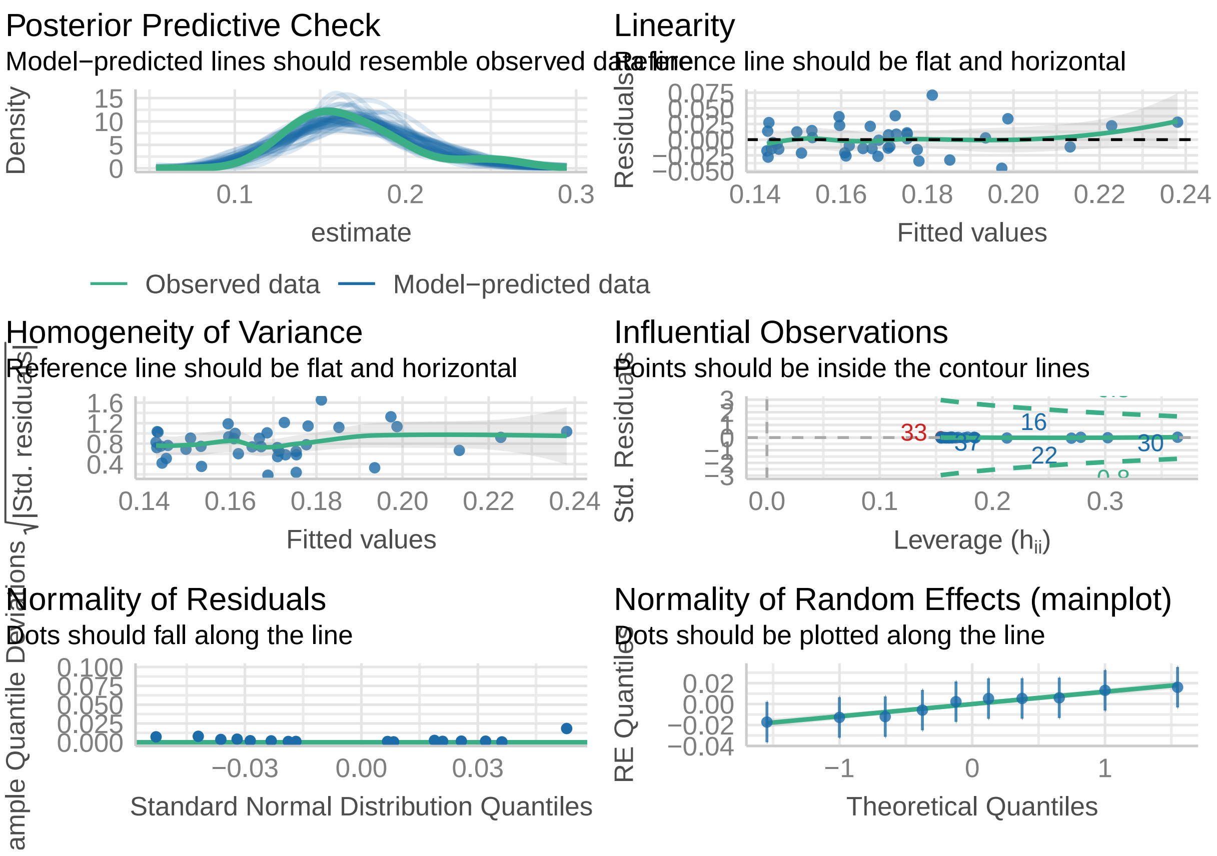


```
ggarrange(
  a[[1]],
  a[[2]],
  a[[3]],
  a[[4]])
```

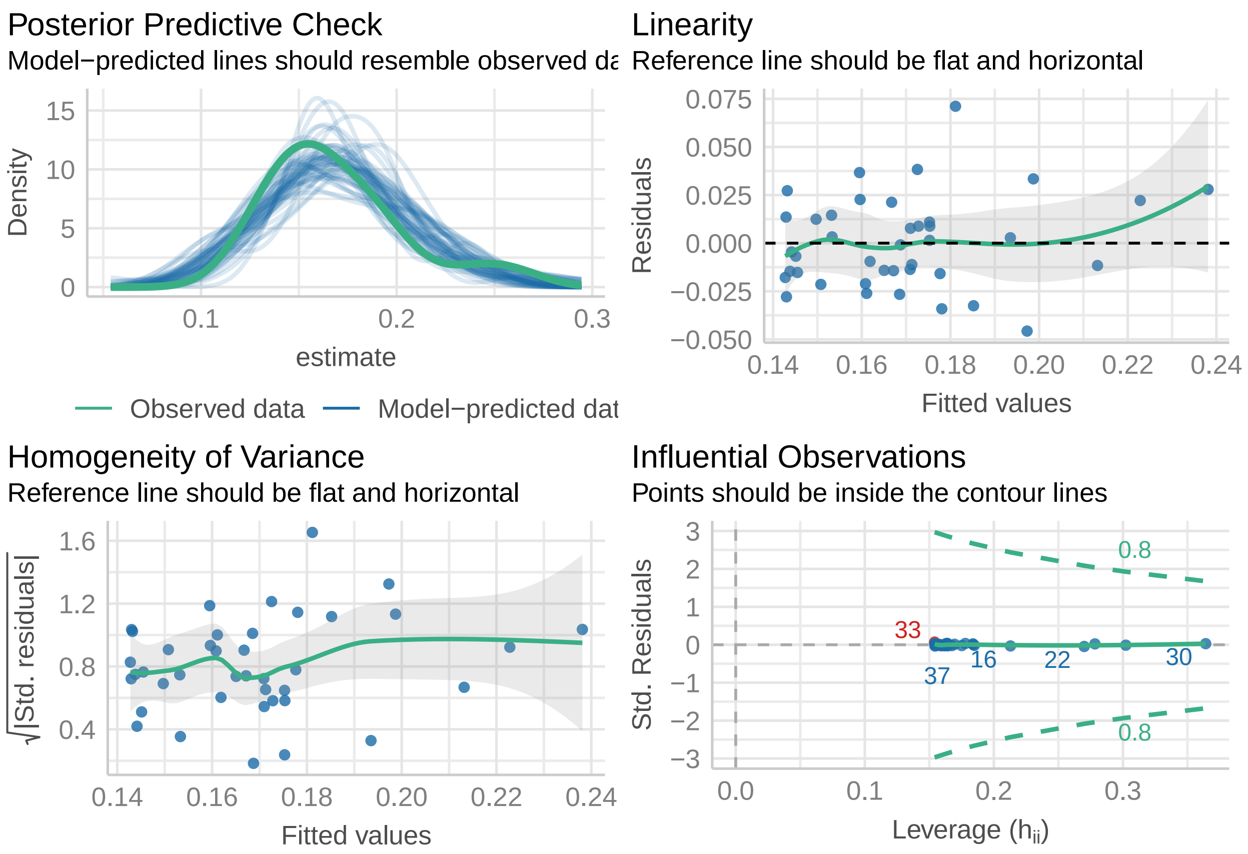

Summary

```
summary(mod)
```

```
## Linear mixed model fit by REML. t-tests use Satterthwaite's method [
## lmerModLmerTest]
## Formula: "estimate ~ f_b + (1|mainplot)"
##    Data: df.1
##
## REML criterion at convergence: -154.9
##
## Scaled residuals:
##     Min      1Q  Median      3Q     Max
## -1.7554 -0.5904 -0.1049  0.5301  2.7333
##
## Random effects:
##  Groups   Name        Variance  Std.Dev.
##  mainplot (Intercept) 0.0002270 0.01507
##  Residual             0.0006776 0.02603
## Number of obs: 40, groups:  mainplot, 10
##
## Fixed effects:
##              Estimate Std. Error        df t value Pr(>|t|)
## (Intercept)  0.151064   0.007350 15.512004  20.552 1.14e-12 ***
## f_b          0.024877   0.005211 31.520148   4.774 3.95e-05 ***
## ---
## Signif. codes:  0 '***' 0.001 '**' 0.01 '*' 0.05 '.' 0.1 ' ' 1
```

```
##
## Correlation of Fixed Effects:
##     (Intr)
## f_b -0.516
```

Supplemantary Figure S4: Relationship between land use, plant biomass production (Yield, T/ha/y), and soil microbial community (total microbial biomass, fungi biomass, bacterial biomass, and fungal-to-bacterial ratio)

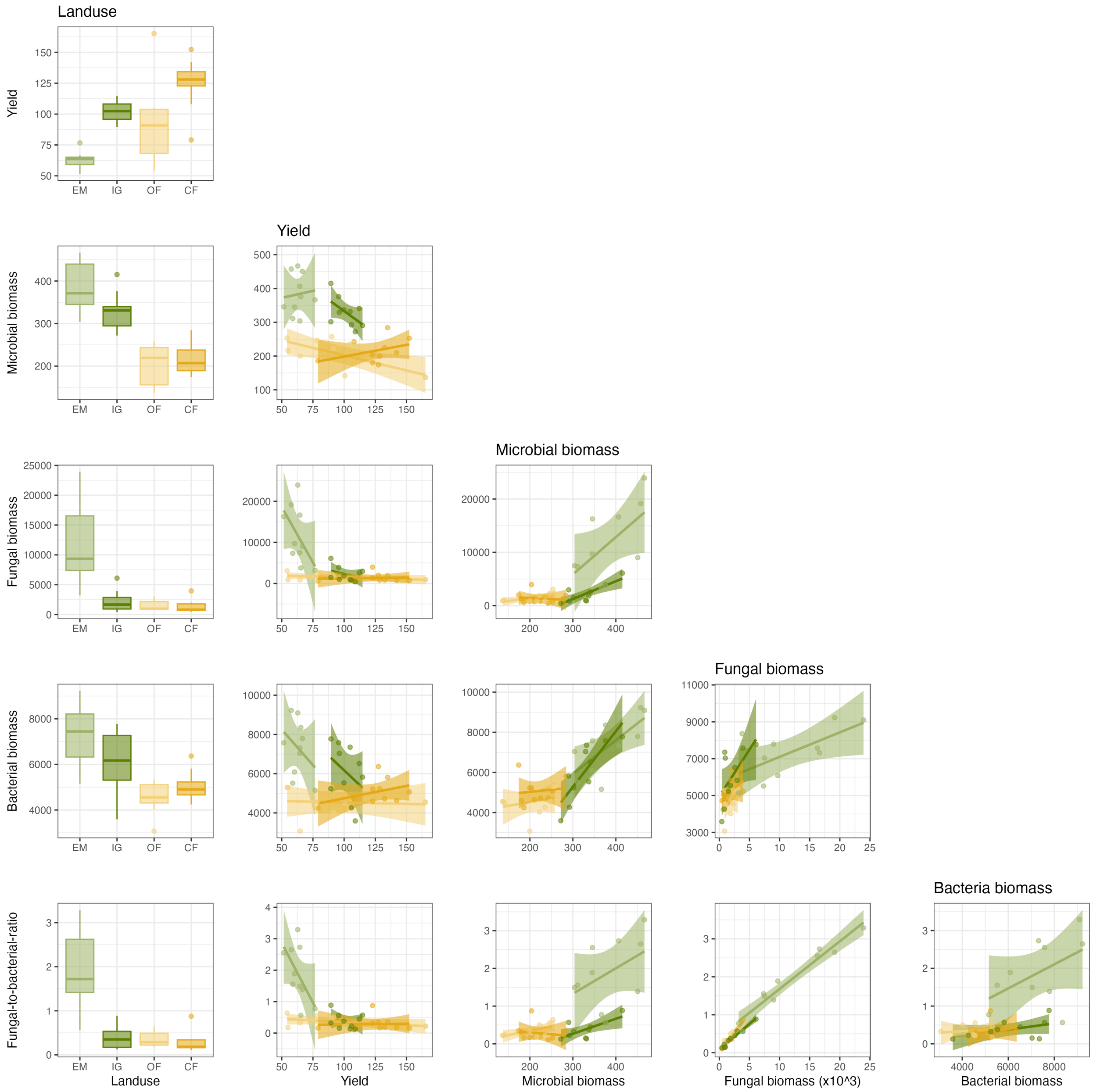

Supplement: Supplementary file 1 — Data S1. [file GCB-31-e70214-s001.pdf]
